# Qingxuan Runmu Yin alleviates dry eye disease via inhibition of the HMOX1/HIF-1 pathway affecting ferroptosis

**DOI:** 10.3389/fphar.2024.1391946

**Published:** 2024-09-11

**Authors:** Jiadi Wang, Yue Liu, Beiting Zong, Shanshan Zhao, Yue Li, Zhirui Zhang, Jing Yao

**Affiliations:** ^1^ The First Affiliated Hospital of Heilongjiang University of Traditional Chinese Medicine, Harbin, China; ^2^ Heilongjiang University of Traditional Chinese Medicine, Harbin, China

**Keywords:** dry eye disease, HMOX1/HIF-1 pathway, ferroptosis, network analysis, Qingxuan Runmu Yin

## Abstract

The prevalence of dry eye disease (DED), a multifactorial ocular surface disease characterized by tear film instability, is increasing yearly. Qingxuan Run Mu Yin (QXRMY) is a traditional Chinese medicine (TCM) consisting of *Radix Rehmanniae, Radix Scrophulariae, Rhizoma Atractylodis macrocephalae, Herba Dendrobii, Flos Lonicerae, Forsythia suspensa, Ophiopogon japonicus, Saposhnikovia divaricata, Radix Platycodi, and Radix Glycyrrhizae.* It has excellent therapeutic effects on dry eye syndrome and a good anti-inflammatory effect on immune-related inflammation. However, the molecular mechanism of Qing Xuan Run Mu Yin in treating dry eye syndrome is largely unknown. The present study used an online database to identify potential target genes of QXRMY for treating DED. The possible mechanisms of these target genes for the treatment of DED were obtained through Gene Ontology (GO) and Kyoto Encyclopaedia of Genes and Genomes (KEGG) databases, Hub genes screened by Cytoscape and intersected with ferroptosis-related genes, and the essential genes were finally obtained based on the results of the analyses. DED cell model and rat model were constructed in this study to validate the critical genes and pathways, and it was confirmed that QXEMY alleviated DED by repressing ferroptosis through inhibiting the HMOX1/HIF-1 pathway. In conclusion, this study integrated network pharmacological analyses and experimental validation to provide an effective method to investigate the molecular mechanism of QXRMY in treating DED.

## 1 Introduction

DED is one of the most prevalent eye diseases worldwide (Marshall and Roach, 2016; Zhu et al., 2014), and the prevalence of this disease increases with the patient’s age (Vehof et al., 2021). DED restricts the patient’s ability to work through symptoms such as irritation, dryness, burning, and impaired visual acuity, as well as severely affecting the patient’s quality of life (Abetz et al., 2011; Aljeaidi et al., 2020; Craig et al., 2017; McDonald et al., 2016) Currently, although the pathogenesis of DED has not been fully elucidated, inflammation, as a result of early innate and adaptive immune responses, is considered to be a critical pathogenetic mechanism in DED and is thought to be a crucial factor in the progression of DED (Kojima et al., 2020; Rhee and Mah, 2017; Shimazaki, 2018). Currently, the Food and Drug Administration (FDA) has approved Cyclosporin, Lifitegrast, and Loteprednol etabonate ophthalmic, three immunomodulatory/anti-inflammatory drugs with immunomodulatory/anti-inflammatory properties, for the treatment of DED (Holland et al., 2019; Pflugfelder and de Paiva, 2017). However, these medications typically require frequent administration, which increases the probability of adverse effects and leads to poor patient compliance (Wirta et al., 2019). Therefore, there is an urgent need to develop safe and effective treatment strategies for DED.

Ferroptosis is a mode of programmed cell death triggered by iron-dependent accumulation of reactive oxygen species as well as lipid peroxides (Dixon et al., 2012; Wu et al., 2019; Xie et al., 2016), and it has been demonstrated in many studies that ferroptosis is associated with a variety of physiopathological processes in different organs of the human body (Hassannia et al., 2019; Li et al., 2020), and ferroptosis is involved in the development of a variety of diseases (Luo et al., 2021; Ni et al., 2022; Tan et al., 2021; Van Coillie et al., 2022; Zhang et al., 2022). Ferroptosis plays a vital role in ophthalmic diseases; for example, in a mouse model of dry age-related macular degeneration (AMD), exposure of receptor cells to all-trans-retinal (atRAL) activates COX2, which further induces Fe^2+^ overload, glutathione (GSH) depletion and mitochondrial Impairment can cause reactive oxygen species (ROS) production, which synergistically promotes lipid peroxidation with ACSL4 activation, thereby causing cellular ferroptosis (Chen et al., 2021), It has been shown (Ogawa, 2015; Sakai et al., 2016) that inhibition of ferroptosis plays a protective role in corneal cells. In summary, ferroptosis may be a crucial therapeutic target for DED.

Based on the theory of “simultaneous treatment of lung and spleen,” QXRMY has an excellent therapeutic effect on DED, and it has been shown to improve ocular surface symptoms, increase tear secretion, prolong tear film rupture time, and maintain tear film stability (Jia et al., 2023; Jing et al., 2014). However, there has been no study to elucidate the molecular mechanism of QXRMY in treating DED.

The mechanism of QXRMY in the treatment of DED was investigated in this study using network pharmacological analysis and experimental validation. Firstly, network pharmacology was used to search for the drug and disease targets of QXRMY. The potential targets of QXRMY for the treatment of DED were screened and subjected to Gene Ontology (GO)/Kyoto Encyclopedia of Genes and Genomes (KEGG) enrichment analyses. It was found that these potential targets were significantly enriched in the pathways of “response to oxidative stress,” “response to metal ion,” etc. The results were summarized as follows. The potential therapeutic targets were significantly enriched in the “response to oxidative stress” and “response to metal ion” pathways. In this study, we hypothesized that QXRMY could inhibit ferroptosis in DED and verified the mechanism of action of QXRMY on DED at cellular and animal levels. The results of this study demonstrated that QXRMY alleviated dry eye by inhibiting ferroptosis through inhibiting the HMOX1/HIF-1 pathway.

## 2 Materials and methods

### 2.1 Screening potential therapeutic targets of QXRMY for the treatment of DED

The HERB database was searched using the HERB online database to collect the drug targets of *Radix Rehmanniae, Radix Scrophulariae, Rhizoma Atractylodis macrocephalae, Herba Dendrobii, Flos Lonicerae, Forsythia suspensa, Ophiopogon japonicus, Saposhnikovia divaricata, Radix Platycodi, and Radix Glycyrrhizae.* The GeneCards (https://www.disgenet.org/home/) and DisGeNET databases (https://www.genecards.org/) were used to search for relevant disease targets with the keyword “dry eye disease”. Finally, the intersection of the drug and disease targets was taken to obtain the potential therapeutic target of QXRMY for treating DED.

### 2.2 Gene ontology and kyoto encyclopedia of genes and genomes

Gene Ontology (GO) terminology and Kyoto Encyclopedia of Genes and Genomes (KEGG) enrichment analyses of potential therapeutic targets were performed using the R package ClusterProfiler (version 3.6.3) (Yu et al., 2012), and the results were visualized.

### 2.3 Protein-protein interaction networks (PPI) construction and hub gene screenings

STRING (version: 10.0, http://www.string-db.org/) database was used to construct a PPI network of proteins coding for potential therapeutic targets, adjusting the confidence level, downloading the tsv file, completing the PPI network visualization using Cytoscape, and extracting the top thirty hub genes with the Hubba plugin in Cytoscape—the top thirty hub genes with the highest MCC scores. Downloaded ferroptosis-related genes in the FerrDB database and intersected them with the previously obtained hub genes.

### 2.4 The establishment of a hyperosmotic-induced human corneal epithelial cell line HCE-2 *in vitro* models of DED

A human SV40 immortalized corneal epithelial cell line (CRL-11135, HCE-2; ATCC, Manassas, VA) were treated with 69 mM NaCl for 24 h (the osmotic pressure of the medium was measured as 450 OsM using a vapor pressure osmometer), i.e., the model and control groups. The medium was IMDM complete medium supplemented with 10% fetal bovine serum, 100 U/mL penicillin, and 100 mg/mL streptomycin, and the medium was changed once in 2–3 days. The cells were cultured in 5% CO_2_ at 37°C in an incubator.

### 2.5 Preparation of drug-containing serum of QXRMY

The wild-type Wistar rats were gavaged with QXRMY (rat dose (g/kg) = adult dose (mg/d)/60 kg)) for 7 consecutive days. One hour after the last gavage, blood was taken from the abdominal vein, serum was isolated under sterile conditions, and heat was inactivated. The cell serum of the treatment group used the above QXRMY-containing serum, and the control group still used fetal bovine serum. Our study protocol was approved by the ethics committees of Heilongjiang University of Chinese Medicine.

### 2.6 Detection of Fe^2+^, malondialdehyde (MDA; a product of lipid peroxidation), GSH, and ROS

Iron assay (Elabscience, China; E-BC-K773-M), MDA assay (AAT Bioquest, United States, 10070), reduced GSH assay (Elabscience, China, E-EL-0026), and ROS assay (AAT Bioquest, United States, 22900) kits were used according to the manufacturer’s instructions. Each group contained four replicates, with two duplicate wells for the blank and standard. Absorbance values were detected using an enzyme marker (BioTek Instruments, Winooski, VT, United States). The Fe^2+^, MDA, GSH, and ROS contents were calculated using the formulas provided in the relevant manufacturers’ instructions.

### 2.7 Apoptosis assays

Using the Annexin V-FITC/PI Apoptosis Detection Kit (Elabscience, Wuhan, China, E-CK-A211), approximately 5 × 10^5^ cells were added to 60 mM dishes and given different treatments. After 24 h of treatment, cells were collected and resuspended in 300 μL of ice-cold 1 × binding buffer and stained with PI and FITC Annexin V for approximately 15 min at 4°C in the dark. The results were analyzed by flow cytometry.

### 2.8 Western blot

Protein blot analysis was routinely performed. HCE-2 was inoculated in culture plates under different conditions. All cells were washed with PBS buffer and lysed with lysis buffer (Beyotime Institute of Biotechnology). Protein concentration was measured using the BCA method (Invitrogen Inc., United States). Equal amounts (30 μg) of proteins were loaded onto a 10% SDS-polyacrylamide gel and separated, then transferred to a PVDF membrane. The membranes were blocked with TBST containing 5% skimmed milk for 1 h at room temperature and washed thrice. The membranes were incubated with primary antibody for blotting at 4°C overnight. The blot was then washed in TBST and set with a secondary antibody for 2 h at room temperature. The blot was washed in TBST. Immunoreactive bands were visualized by chemiluminescence using Enhanced Chemiluminescence Enhanced (GE Healthcare).GAPDH was used as an internal marker. Protein levels were expressed as the ratio of the grey scale value of the target band to the grey scale value of GADPH. All the primary antibodies used are listed as follows:

HMOX1 (Abcam, United States, ab68477)
HIF-1α (Abcam, United States, ab1)
GADPH (Abcam, United States, ab181602)

### 2.9 Viability assay

The CCK-8 assay was performed per the manufacturer’s protocol to assess cell viability. Briefly, cells were inoculated in 96-well plates and exposed to a conditioned medium. Then, 100 μL of a medium and CCK-8 solution mixture is added to each plate well. The plates were then incubated for 1–2 h (37°C, 5% CO_2_). The absorbance at 450 nm was measured using an enzyme marker (BioTek Instruments, Winooski, VT, United States).

### 2.10 Quantitative RT-PCR (qRT-PCR)

According to the manufacturer’s instructions, total RNA was extracted from HCEC or intact corneas using the RNeasy Mini Kit (Qiagen). A spectrophotometer (NanoDrop ND-1000; Thermo Fisher Scientific, Waltham, MA, United States) was used. The cDNA was synthesized with PrimeScript RT Master Mix (TAKARA BIO INC, Shiga, Japan) and then amplified in a Light Cycler 480 real-time fluorescent quantitative PCR system using SYBR Green Supermix (Bio-Rad Laboratories, Inc.) for Amplification. The pre-designed primers are listed as follows:

GPX4 Forward: ACA CCG TCT CTC CAC AGT TC.
   Reverse: ACG CTG GAT TTT CGG GTC TG.
ACSL4 Forward: CCT​GAG​GGG​CTT​GAA​ATT​CAC.
   Reverse: GTT​GGT​CTA​CTT​GGA​GGA​ACG.
TFRC Forward: GTT​TCT​GCC​AGC​CCC​TTA​TTA​T.
   Reverse: GCA​AGG​AAA​GGA​TAT​GCA​GCA
GAPDH Forward: AGG​TCG​GTG​TGA​ACG​GAT​TTG.
   Reverse: GGG​GTC​GTT​GAT​GGC​AAC​A.

### 2.11 Animals and treatment

The 7-week-old male Wistar rats were purchased from GENET-MED (Jilin, China). The rats were anesthetized by intraperitoneal injection of ketamine (75 mg/kg) and xylazine (10 mg/kg). After anesthesia, the lacrimal gland was surgically removed. The animal experiments were approved by the Institutional Animal Care and Use Committee (2023090601).

### 2.12 Tear secretion and sodium fluorescein staining

Tear secretion in rats was measured by the phenol red silk method. The filament was placed on the lateral eye for 15 s, and the length of the wet red filament was recorded in millimeters. 5 μL (1%) of dry fluorescein was placed on the surface of the rat eye to observe corneal epithelial destruction. Photographs of the eyes were taken with a digital camera.

## 3 Results

### 3.1 Joint target acquisition for QXRMY-DED

HERB online database (Fang et al., 2021) was used to search and collect the drug targets of DED, Xuan Shen, Atractylodes macrocephala, Dendrobium, Honeysuckle, Forsythia, Maitake, Fenghuang, Platycodonopsis, and Glycyrrhiza glabra, and a total of 278 target genes were obtained by combining the drug targets of the ten traditional Chinese medicines. A total of 4,116 disease targets were searched by taking “DED” as the keyword in the GeneCards. Using “dry eye disease” as the keyword, the GeneCards and DisGeNET databases were searched for related disease targets, and 4,116 disease targets were identified by removing duplicates. Finally, the intersection of disease targets with drug targets resulted in a total of 145 hits (Supplementary Table S1), which were identified in this study as potential.

### 3.2 Therapeutic targets for QXRMY in the treatment of DED

The HERB online database (Fang et al., 2021) was searched to collect the drug targets of Radix Rehmanniae, Radix Scrophulariae, Rhizoma Atractylodis macrocephalae, Herba Dendrobii, Flos Lonicerae, Forsythia suspensa, Ophiopogon japonicus, Saposhnikovia divaricata, Radix Platycodi, and Radix Glycyrrhizae, and a total of 278 target genes were obtained by combining the drug targets of the ten traditional Chinese medicines. DED″ as the keyword, we searched the GeneCards and DisGeNET databases for related disease targets, and there were a total of 4,116 disease targets after de-aggregating and removing duplicates, finally, the intersection of disease targets with drug targets resulted in a total of 145 hits. This study described these targets as potential therapeutic targets for QXRMY in treating DED.

### 3.3 Gene ontology and kyoto encyclopedia of genes and genomes

To further investigate the functions of the above potential therapeutic targets, 145 genes were subjected to GO and KEGG pathway enrichment analyses in this study, with a screening threshold of p.adjust < 0.05, and the top 20 pathways were visualized by using ggplot (Figures A–D), we found that these potential therapeutic targets were significantly enriched in the paths of “response to oxidative stress,” “response to metal ion,” etc. KEGG pathway enrichment analyses showed that the most prominent ones were PI3K-Akt signaling, and the most prominent ones were PI3K-Akt signaling. Oxidative stress,” “response to metal ion,” and KEGG pathway enrichment analysis showed that the most prominent pathways were the PI3K-Akt signaling pathway and HIF-1 signaling pathway. Previous studies have shown (Fujiki et al., 2019; Luo et al., 2018) that response to oxidative stress and response to metal ions play critical roles in ferroptosis. Meanwhile, DED is accompanied by ferroptosis (Scarpellini et al., 2023); based on this result, we hypothesized that QXRMY produces a therapeutic effect on DED through ferroptosis.

**FIGURE 1:**
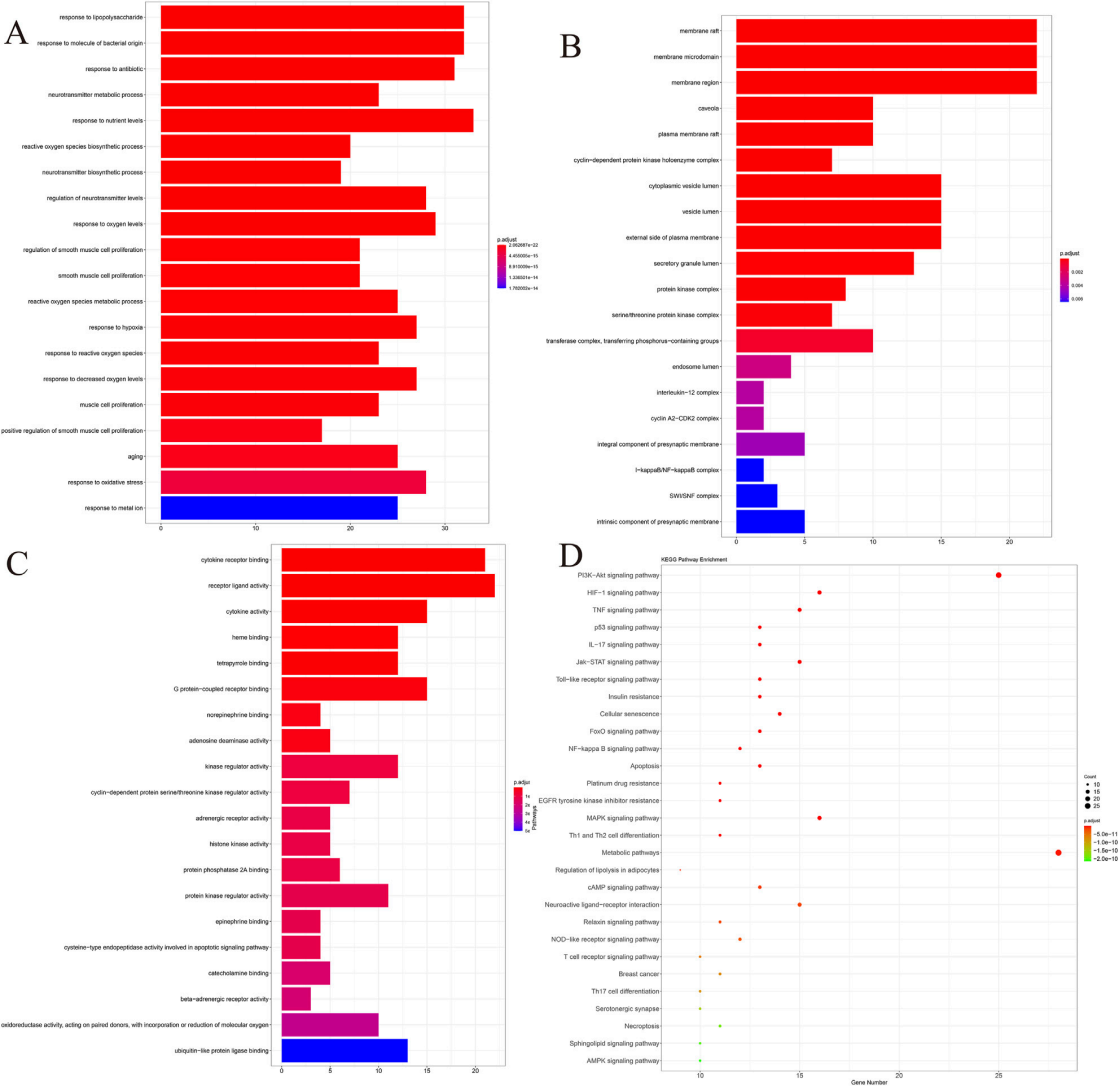
Co-target genes were analyzed by GO and KEGG. **(A)** BP. **(B)** CC. **(C)** MF. **(D)** KEGG. P-value of the item in the upper left corner, the gene ratio of the item in the upper right corner, the interaction between the items in the lower left corner, and the detailed list of the item in the lower right corner.

### 3.4 QXRMY suppresses ferroptosis in DED

**FIGURE 2:**
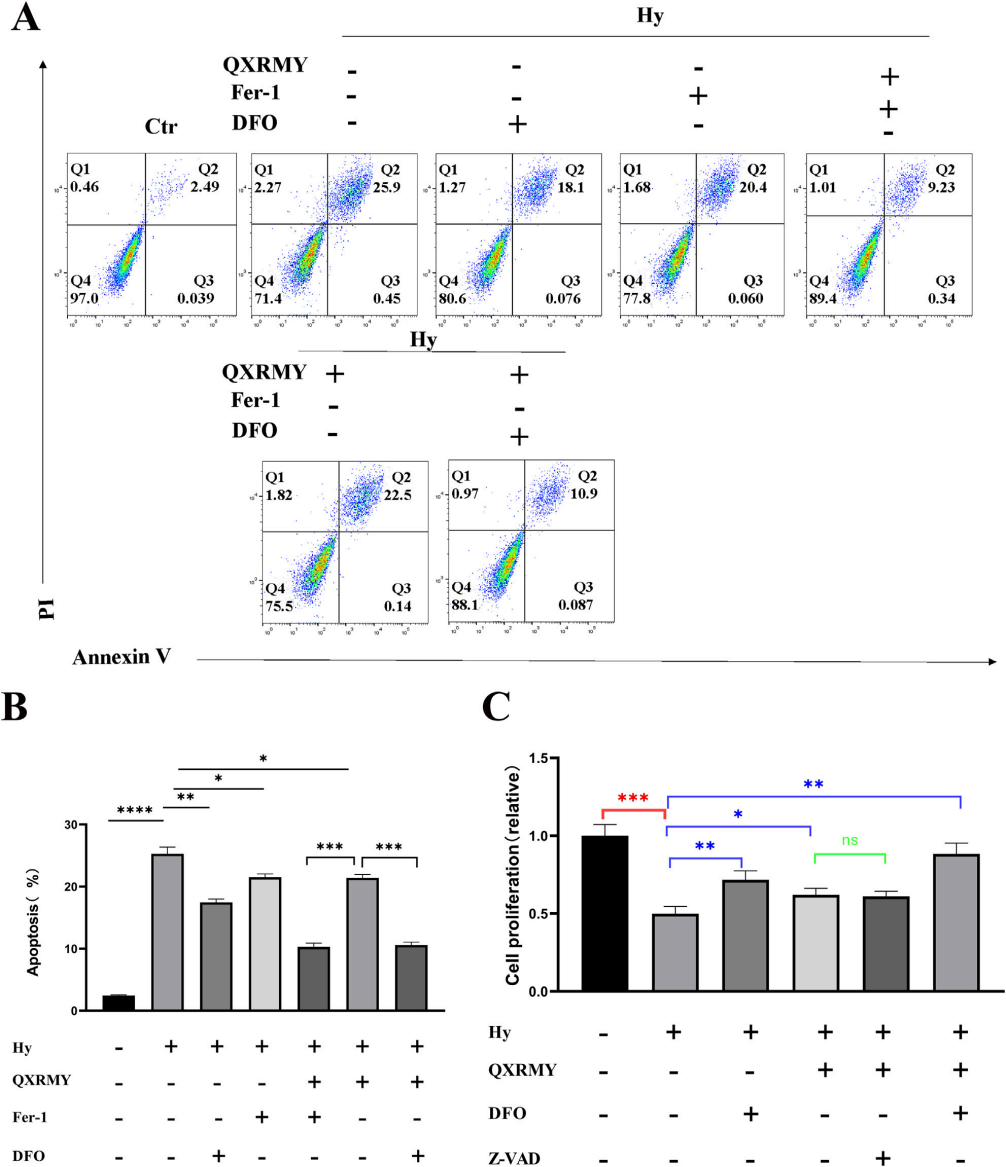
**(A, B)** Apoptosis was detected using PI and annexin-V. **(C)** Examination of cellular activity using CCK8. Error bars indicate SEM, *p < 0.05, **p < 0.01, ***p < 0.001 ****p < 0.0001 by *t*-test.n = 3.

To investigate whether QXRMY affects ferroptosis in DED, HCE-2 cells were exposed to hyperosmolarity, and a DED model was established *in vitro*. Flow cytometry was performed to detect apoptosis, and as shown in Figure 2A, QXRMY, ferroptosis inhibitor deferoxamine (DFO) and Ferrostatin-1 (Fer-1) were able to inhibit apoptosis in the hypertonic group. In addition, apoptosis was significantly reduced when DFO or Fer-1 was combined with QXRMY compared to the group using QXRMY alone.

**FIGURE 3:**
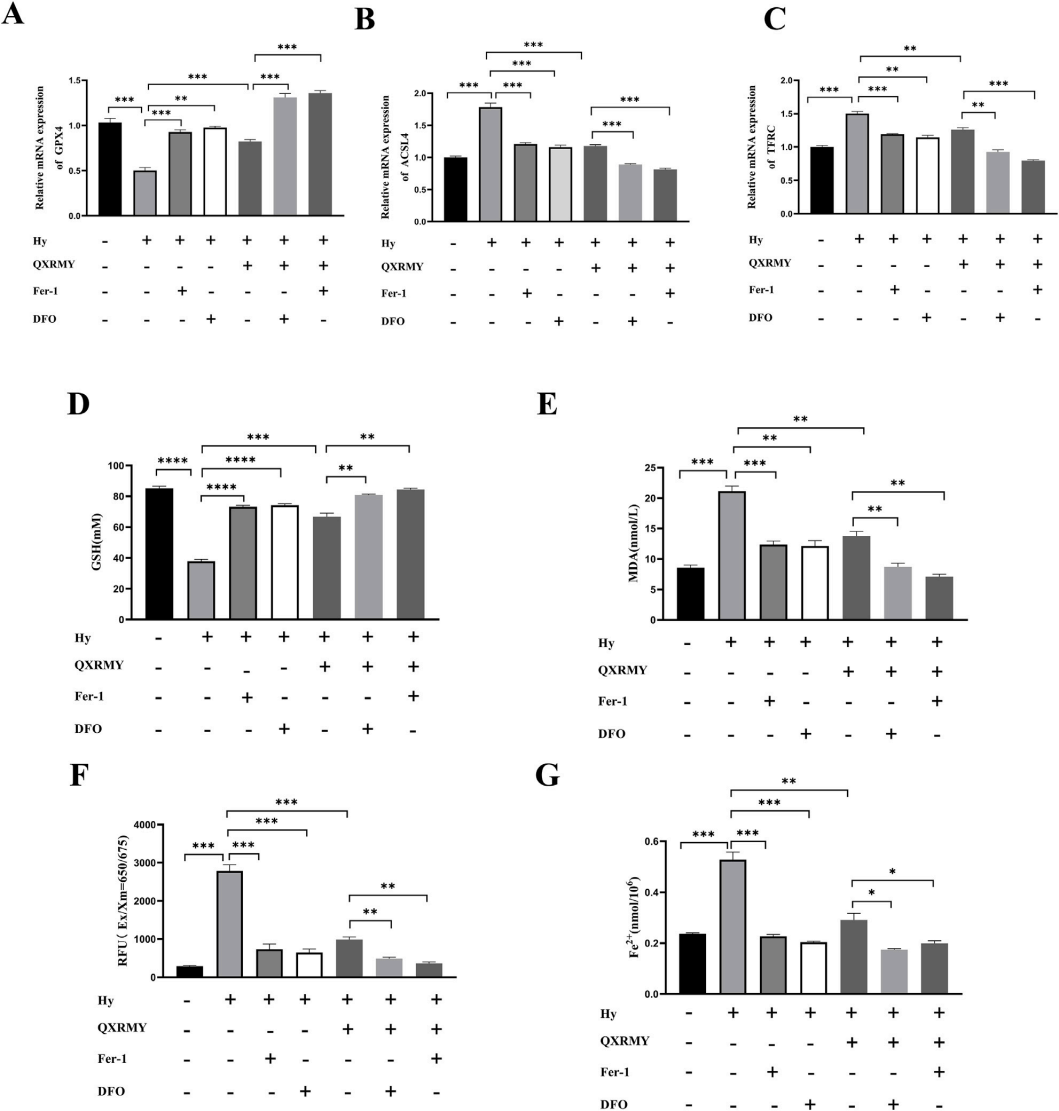
**(A**–**C)** Expression of mRNA for ferroptosis-related genes was compared in different groups. **(D**–**G)** Variation in glutathione (GSH), malonic dialdehyde (MDA), reactive oxygen species (ROS), and Fe^2+^ content between groups. Error bars indicate SEM, *p < 0.05, **p < 0.01, ***p < 0.001 ****p < 0.0001 by *t*-test.n = 3.

To determine whether ferroptosis is the predominant form of cell death leading to cell death in the DED model, cells were treated in this study with pan-caspase inhibitor (Z-VAD-FMK), DFO, or Fer-1 in combination with QXRMY. Compared with the grouping of QXRMY alone, the combination of DFO or Fer-1 with QXRMY increased cell viability. In contrast, the combination of Z-VAD-FMK and QXRMY did not significantly alter the cell viability status. The present study showed that QXRMY increased the mRNA expression of the negative regulator of ferroptosis (GPX4) (Figure 3A) and decreased the mRNA expression of positive regulators (TFRC and ACSL4) in hypertonic HCE-2 cells (Figures 3B, C) by Q-PCR assay.

In addition, this study found that adding QXRMY rescued hypertonicity-induced oxidative stress (Figures 3D–F) and reduced total Fe^2+^ content in cells (Figure 3G). As we predicted, Fer-1 pretreatment with DFO reduced ferroptosis in the DED model (Figures 3A–G) and showed a synergistic relationship with QXRMY. These data demonstrate that QXRMY inhibits hyperosmolarity-induced ferroptosis in HCE-2 cells.

### 3.5 QXRMY alleviates DED by inhibiting ferroptosis by inhibiting the HMOX1/HIF-1 pathway

**FIGURE 4:**
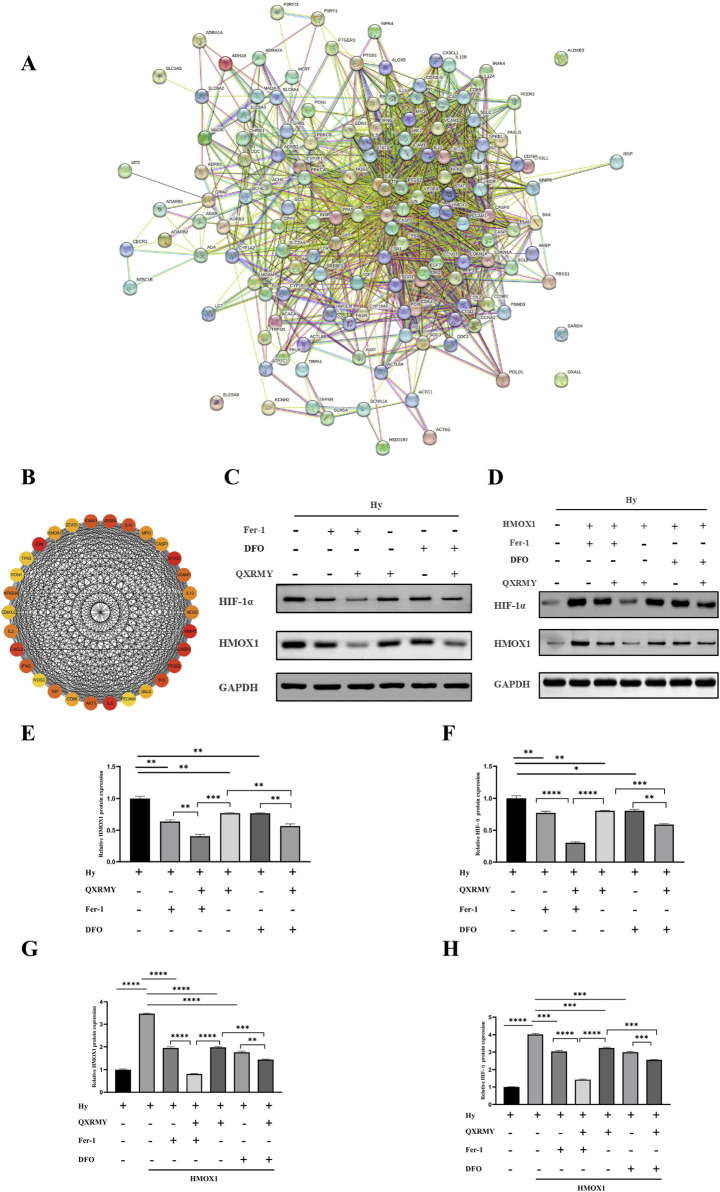
**(A)** Network of essential genes generated with the STRING database. The yellow line represents text mining evidence in the PPI network, and the black line represents co-expressed proteins. **(B)** list of top 30 hub genes explored by CytoHubba. **(C–H)** Western blot analysis was performed to detect the expression of HMOX1 and HIF-1 protein. Error bars indicate SEM, *p < 0.05, **p < 0.01, ***p < 0.001 ****p < 0.0001 by *t*-test.n = 3.

The protein interaction network was constructed in the STRING database (Figure 4A), and the protein interaction data were imported into Cytoscape. Maximal clique centrality (MCC) was calculated for the interaction network using the Hubba plugin, and the top 30 genes with the highest scores were extracted (Figure 4B). Three essential genes were obtained by taking the intersection of the 30 hub genes with ferroptosis-related genes: PTGS2; TP53; and HMOX1. According to our study, HMOX1 was mainly enriched in response to metal ions, and HMOX1 is a critical positive regulator of ferroptosis. According to the KEGG pathway enrichment results, HMOX1 plays a vital role in the HIF-1 signaling pathway and is an essential regulator. Based on the above results, we found that the HMOX1/HIF-1 s pathway may have a critical role in the treatment of DED by QXRMY, so we proposed the hypothesis that QXRMY alleviates DED by inhibiting HMOX1/HIF-1 pathway.

Based on Western blotting results (Figures 4C, E, F), QXRMY, DFO, and Fer-1 rescued the hyperosmolarity-induced increases in HMOX1 and HIF-1 expression. DFO or Fer with QXRMY significantly inhibited the increase in HMOX1 and HIF-1 expression compared with the group using QXRMY alone.

**FIGURE 5:**
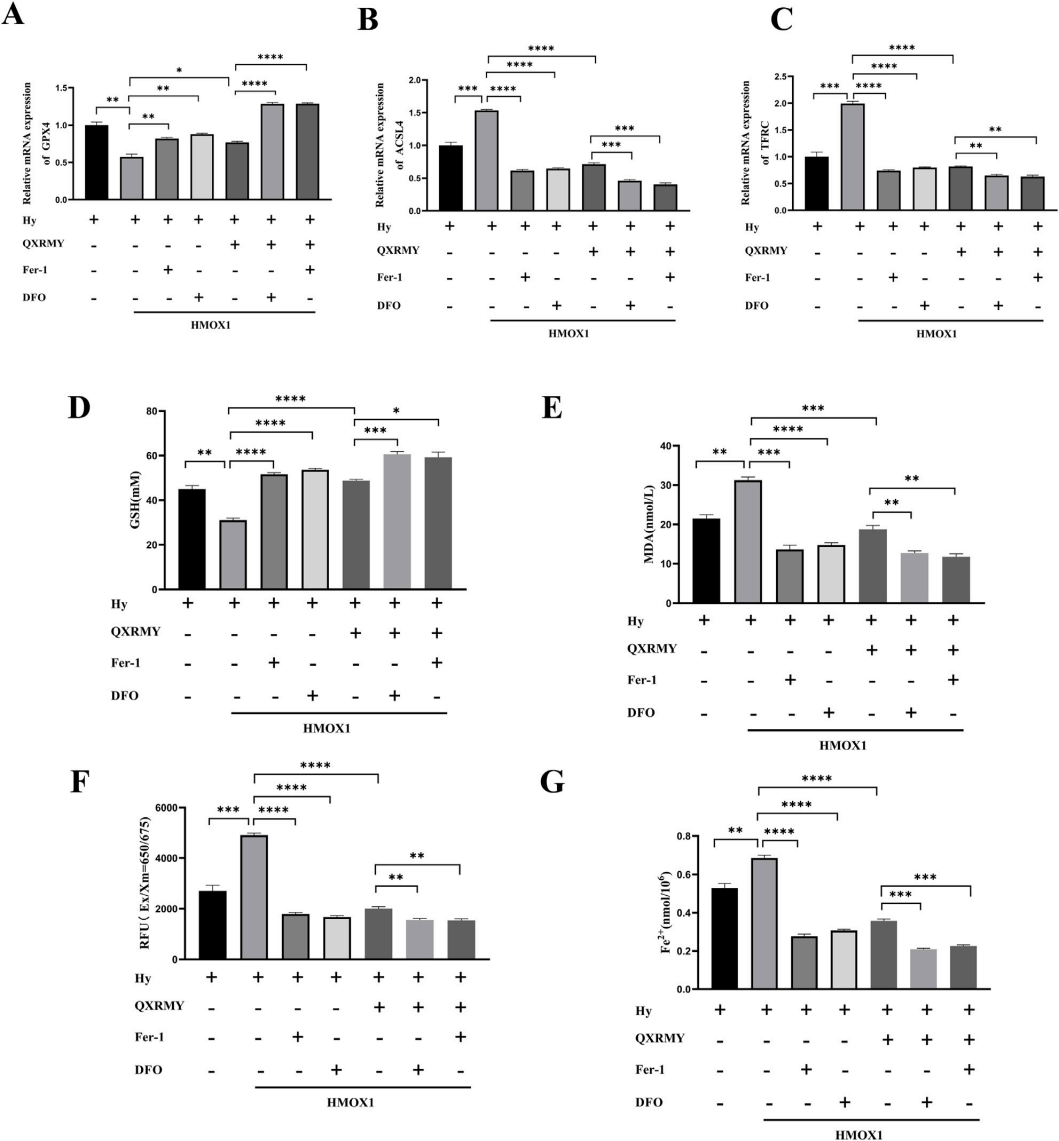
**(A**–**C)** mRNA expression for ferroptosis-related genes was compared in different groups. **(D**–**G)** Variation in glutathione (GSH), malonic dialdehyde (MDA), reactive oxygen species (ROS), and Fe^2+^ content between groups. Error bars indicate SEM, *p < 0.05, **p < 0.01, ***p < 0.001 ****p < 0.0001 by *t*-test.n = 3.

To further test the hypothesis that QXRMY inhibits ferroptosis through inhibition of the HMOX1/HIF-1 pathway, we produced an HMOX1 overexpressing hypertonic HCE-2 cell model. In this model, QXRMY, DFO, and Fer-1 rescued the increase in HMOX1 and HIF-1 expression caused by HMOX1 overexpression (Figures 4D, G, H). DFO or Fer-1 with QXRMY significantly inhibited the increase in HMOX1 and HIF-1 expression compared to the group with QXRMY alone (Figures 4D, G, H). The mRNA expression of the negative regulator of ferroptosis (GPX4) was decreased (Figure 5A) while the mRNA expression of the positive regulators (TFRC and ACSL4) was increased (Figures 5B, C). The addition of QXRMY alleviated the hypertonicity-induced oxidative stress and decreased the total Fe2 content of the cells (Figures 5D–G). However, the combination of QXRMY and Fer or DFO attenuated these results (Figures 5D–G). The above results suggest that QXRMY inhibits ferroptosis by inhibiting the HMOX1/HIF-1 pathway and has a protective effect on hypertonic HCE-2 cells.

### 3.6 Improvement of DED and inhibition of ferroptosis by QXRMY in rats

**FIGURE 6:**
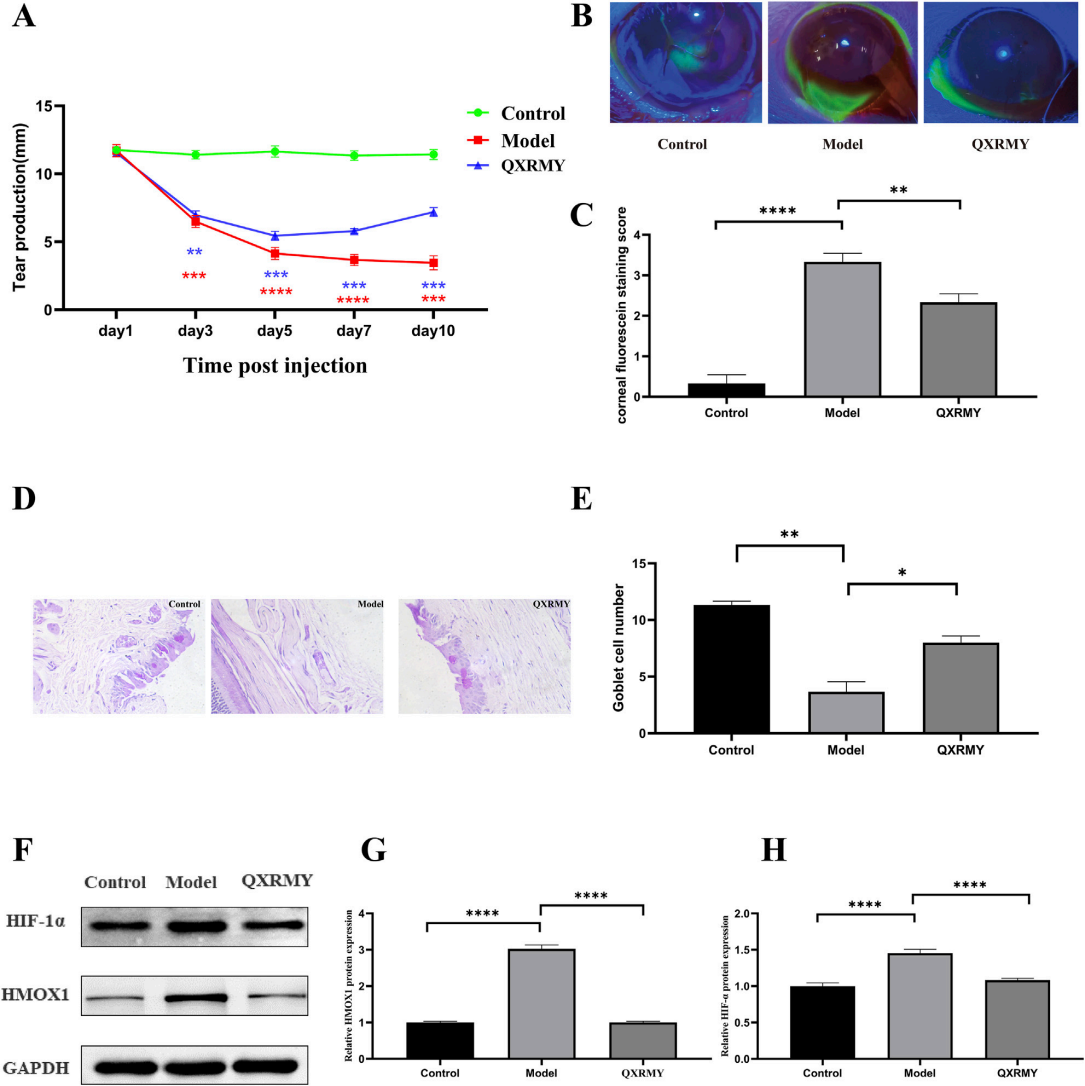
**(A)** Simultaneous modeling and post-administration, the tear secretion of the rats was examined on days 3, 5, 7, and 10. **(B**, **C)** Following simultaneous modeling and treatment of rats, corneas were stained with sodium fluorescein on day 5, and data analyzed, **(D, E)** PAS staining of rat conjunctiva was performed to calculate the number of rat conjunctival goblet cells. **(F**–**H)** Western blot analysis was performed to detect the expression of HMOX1, HIF-1 protein. Error bars indicate SEM, *p < 0.05, **p < 0.01, ***p < 0.001 ****p < 0.0001 by *t*-test.n = 6.

For further elucidation of the role of QXRMY drink in DED, this study examined tear secretion in DED rats infused with 5 μL of 0.2% BAC solution. Compared with the DED group, QXRMY significantly reversed the reduction of tear secretion in DED rats (Figure 6A). QXRMY also rescued corneal breakage in DED rats (Figures 6B, C). Meanwhile, according to the PAS staining results of the conjunctival epithelium, QXRMY showed a protective effect on the cup cells of the conjunctival epithelium of DED rats (Figures 6D, E).

**FIGURE 7:**
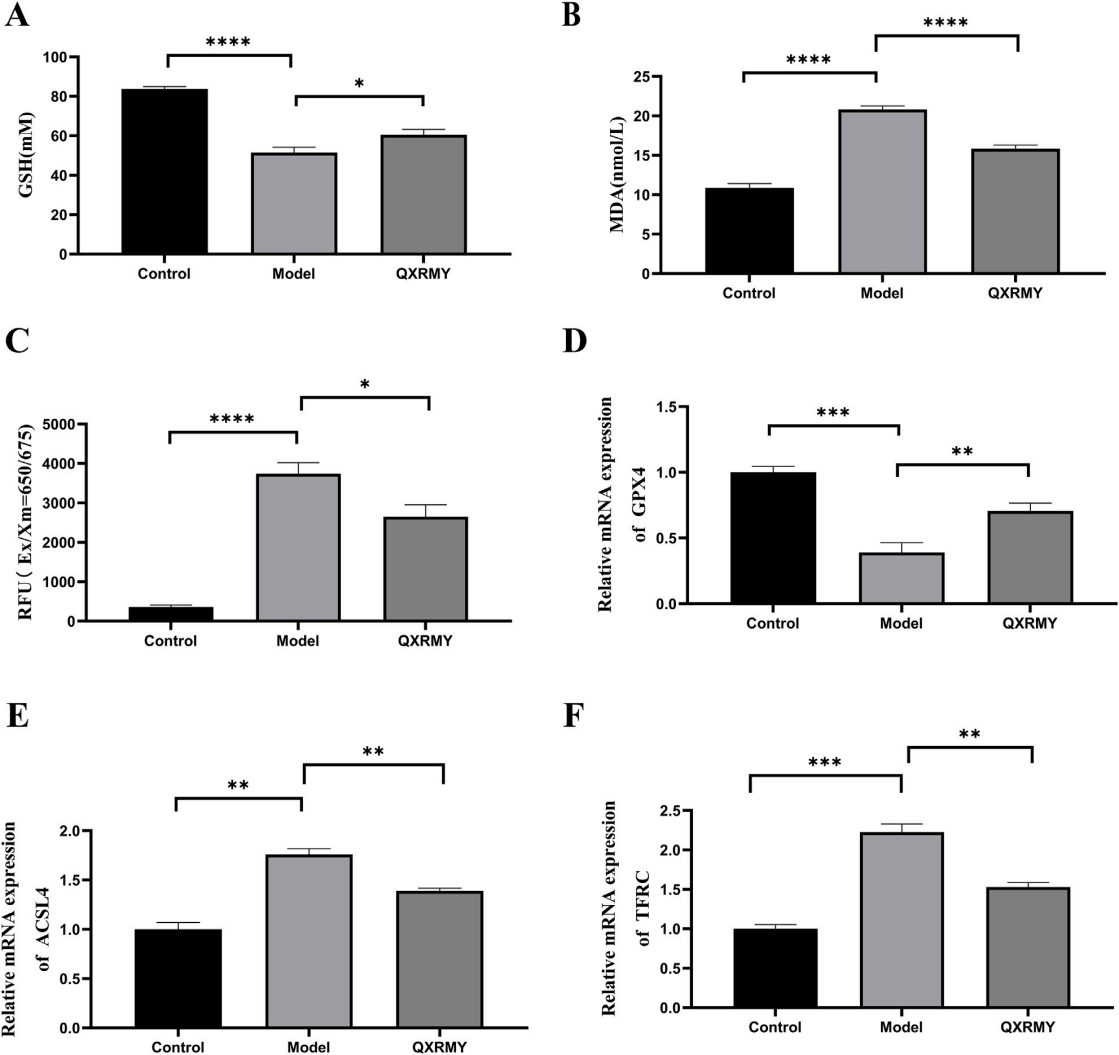
**(A**–**C)** variation in glutathione (GSH), malonic dialdehyde (MDA), reactive oxygen species (ROS), and Fe^2+^ content between groups. **(D**–**F)**. Expression of mRNA for ferroptosis-related genes was compared in different groups. Error bars indicate SEM, *p < 0.05, **p < 0.01, ***p < 0.001 ****p < 0.0001 by *t*-test.n = 6.

In addition, corneal HMOX1 and HIF-1α expressions were increased in DED rats compared with those in the standard group, but the QXRMY group eliminated the effects of DED on HMOX1 and HIF-1α expressions (Figures 6F–H). On the other hand, QXRMY reversed DED-induced oxidative stress (Figures 7A–C), while mRNA expression of negative regulator of corneal ferroptosis (GPX4) was increased, and mRNA expression of positive regulators (TFRC and ACSL4) was decreased in DED rats gavaged with QXRMY (Figures 7D–F). These data suggest that QXRMY can inhibit ferroptosis and alleviate DED symptoms.

## 4 Discussion

DED has a complex pathological process involving apoptosis, oxidative stress, etc. The drugs currently used in the clinic all target a single pathological mechanism and cannot achieve the desired therapeutic state. In contrast, the Chinese herbal medicine compound contains multiple therapeutic components that affect various drug targets. QXRMY is a traditional Chinese medicine formula consisting of 10 botanicals, i.e., *Radix Rehmanniae, Radix Scrophulariae, Rhizoma Atractylodis macrocephalae, Herba Dendrobii, Flos Lonicerae, F.orsythia suspensa, O.phiopogon japonicus, S.aposhnikovia divaricata, Radix Platycodi, and Radix Glycyrrhizae.* QXRMY has been shown to have therapeutic effects on DED (Jia et al., 2023; Jing et al., 2014). However, the underlying mechanism of its treatment of DED is unclear, and it needs to be investigated to determine the mechanism of QXRMY to improve DED. This study used network pharmacology and bioinformatics analyses to predict the drug targets and the molecular mechanisms of QXRMY to treat DED. With the results of the analyses, this study proposed the hypothesis that QXRMY alleviates DED by inhibiting ferroptosis by inhibiting the HMOX1/HIF-1 pathway.

In this study, a total of 278 drug target genes were screened based on network pharmacology and intersected with DED disease targets to obtain potential therapeutic targets.GO, KEGG and PPI network analyses showed that the common target genes were focused on oxidative stress and iron metabolism, which were closely related to ferroptosis. Previous studies have shown (Fujiki et al., 2019; Luo et al., 2018) that response to oxidative stress and response to metal ions play critical roles in ferroptosis. Meanwhile, DED is accompanied by ferroptosis (Scarpellini et al., 2023); based on this result, we hypothesized that QXRMY produces a therapeutic effect on DED by affecting iron death.

Thus, this study focused on the ferroptosis-related targets of QXRMY for treating DED. Among the three essential genes obtained subsequently, HMOX1 is a critical positive regulator of ferroptosis (Wu et al., 2022), and according to the KEGG pathway enrichment results, HMOX1 acts as an essential regulator in the HIF-1 pathway. Therefore, we focused on the HMOX1/HIF-1 pathway to explore the mechanism of QXRMY drink for DED.

It has been shown that ferroptosis is a critical mechanism in the etiological pathology of DED (Lovatt et al., 2020; Zuo et al., 2022). In the present study, we constructed a dry eye cell model using 69 mM NaCl-treated HCE-2 cells to determine the effects of QXRMY on ferroptosis and ferroptosis. All data showed that QXRMY reduced apoptosis, increased cell activity, and harmed ferroptosis in the DED cell model. Our data showed that QXRMY exhibited synergistic effects with Fer-1, an inhibitor of ferroptosis, and rescued HMOX1 overexpression-induced cell death and ferroptosis. In the present study, a rat model of DED was constructed, and according to the experimental results, QXRMY could reduce the symptoms of DED and reverse ferroptosis caused by DED.

In summary, the effects of QXRMY on DED and ferroptosis were determined in this study. The pharmacological effects of QXRMY on DED and ferroptosis were rationally and comprehensively elaborated in this study through network analysis and further combined with a series of experiments; it was proved that QXRMY alleviated DED by inhibiting ferroptosis through inhibiting the HMOX1/HIF-1 pathway. Finally, this study combined network analysis and experimental validation from multiple perspectives to provide a basis for treating DED with QXRMY.

## Data Availability

The raw data supporting the conclusions of this article will be made available by the authors, without undue reservation.
