# Supplementary figures and images for: Qingxuan Runmu Yin alleviates dry eye disease via inhibition of the HMOX1/HIF-1 pathway affecting ferroptosis
Source: Front Pharmacol. 2024 Sep 11;15:1391946. doi: 10.3389/fphar.2024.1391946 (PMC11425584; doi:10.3389/fphar.2024.1391946)

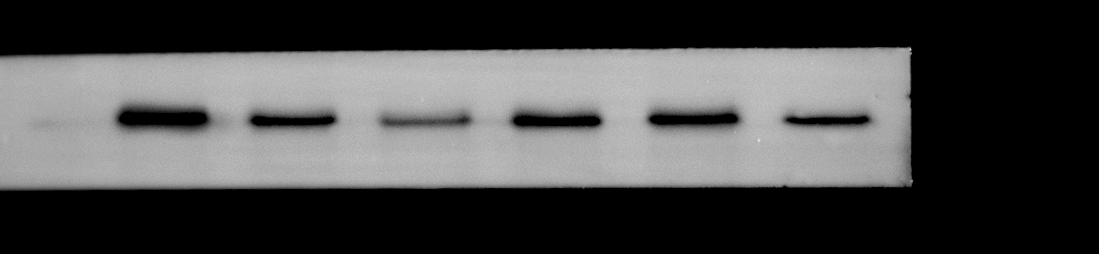

Supplement: Supplementary file 3 [file DataSheet9.ZIP › raw data/2.1.1/1.HMOX1 (2).tif]

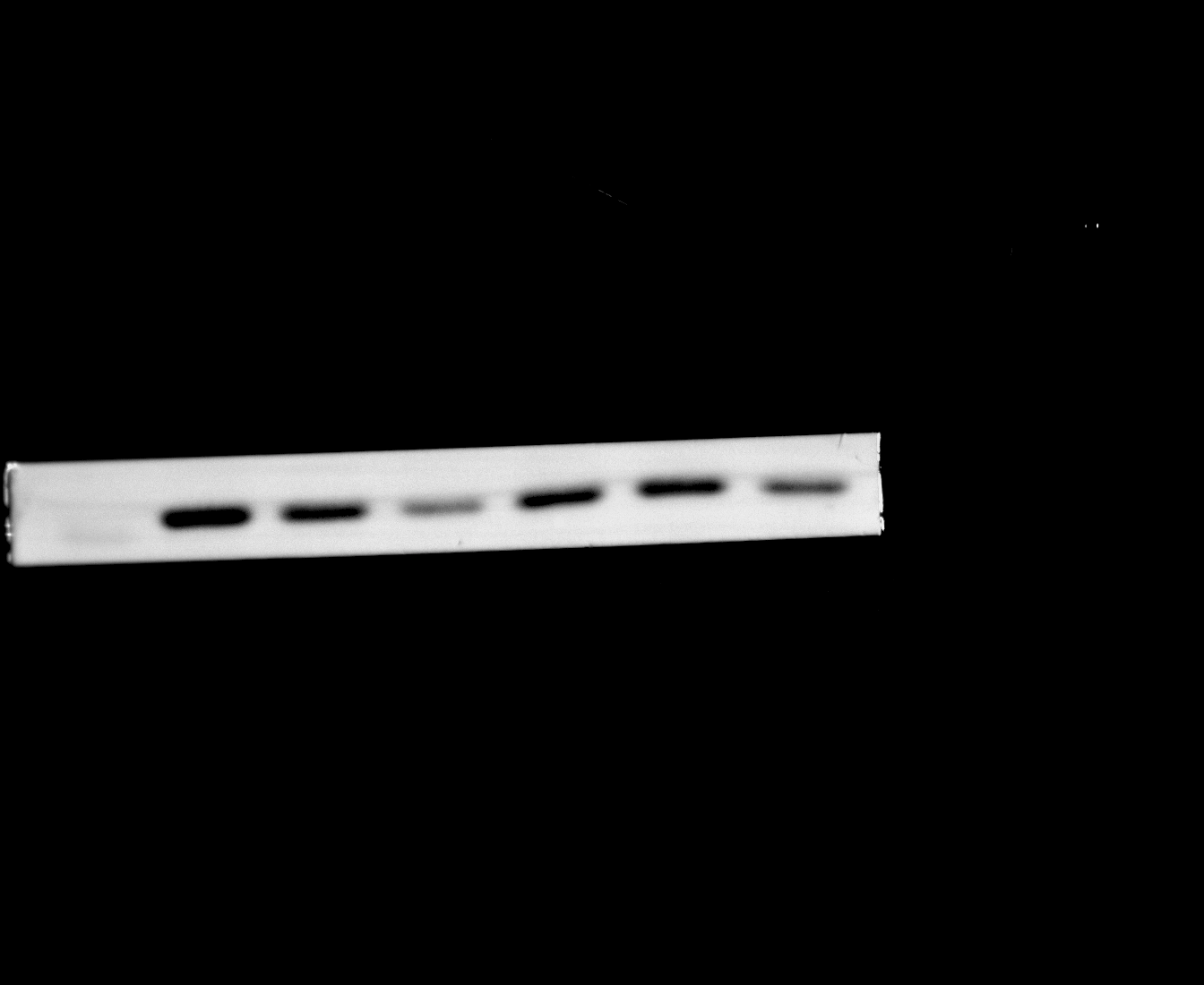

Supplement: Supplementary file 3 [file DataSheet9.ZIP › raw data/2.1.1/2.HIF-1a┴ (2).tif]

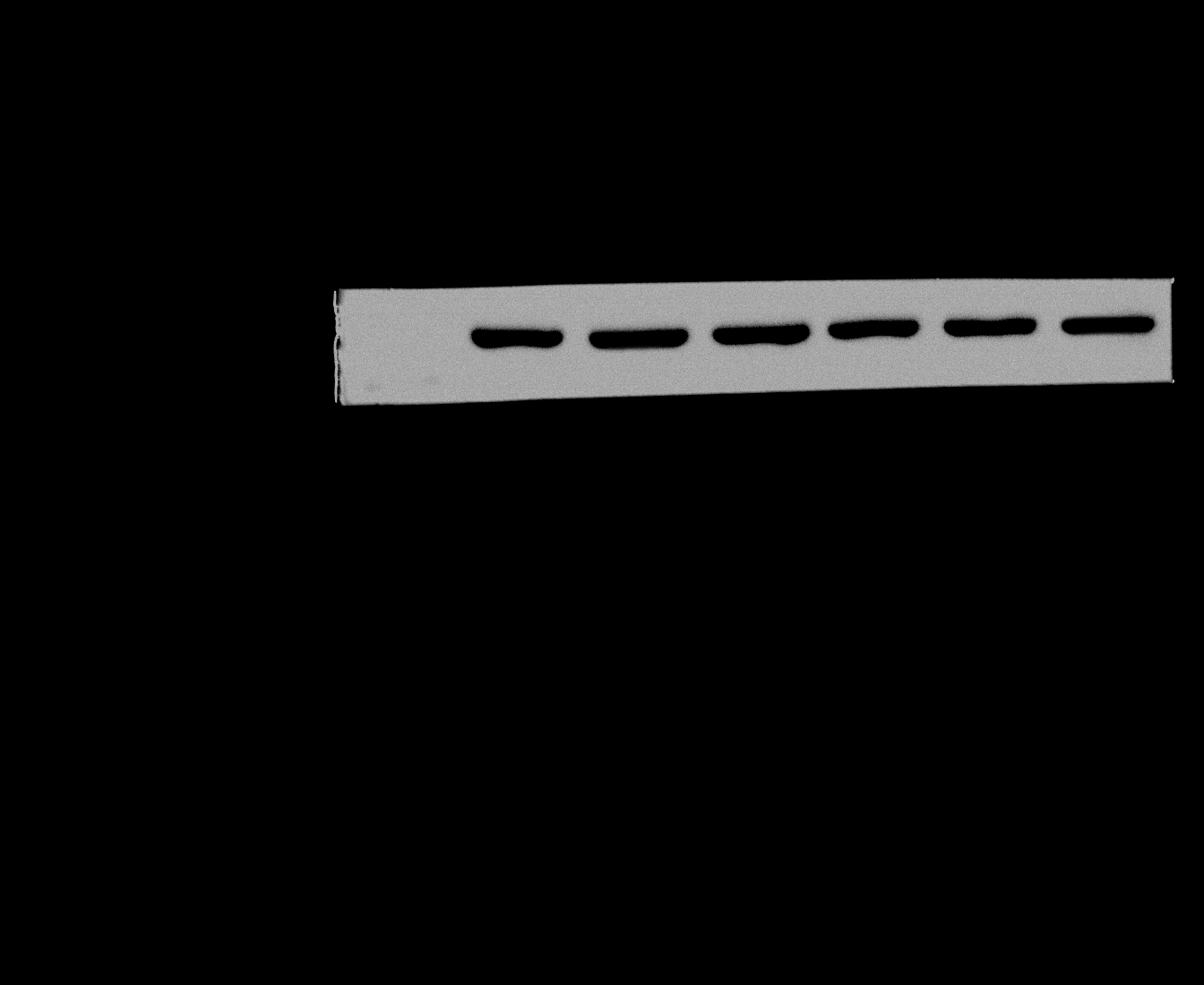

Supplement: Supplementary file 3 [file DataSheet9.ZIP › raw data/2.1.1/─┌▓╬ (2).tif]

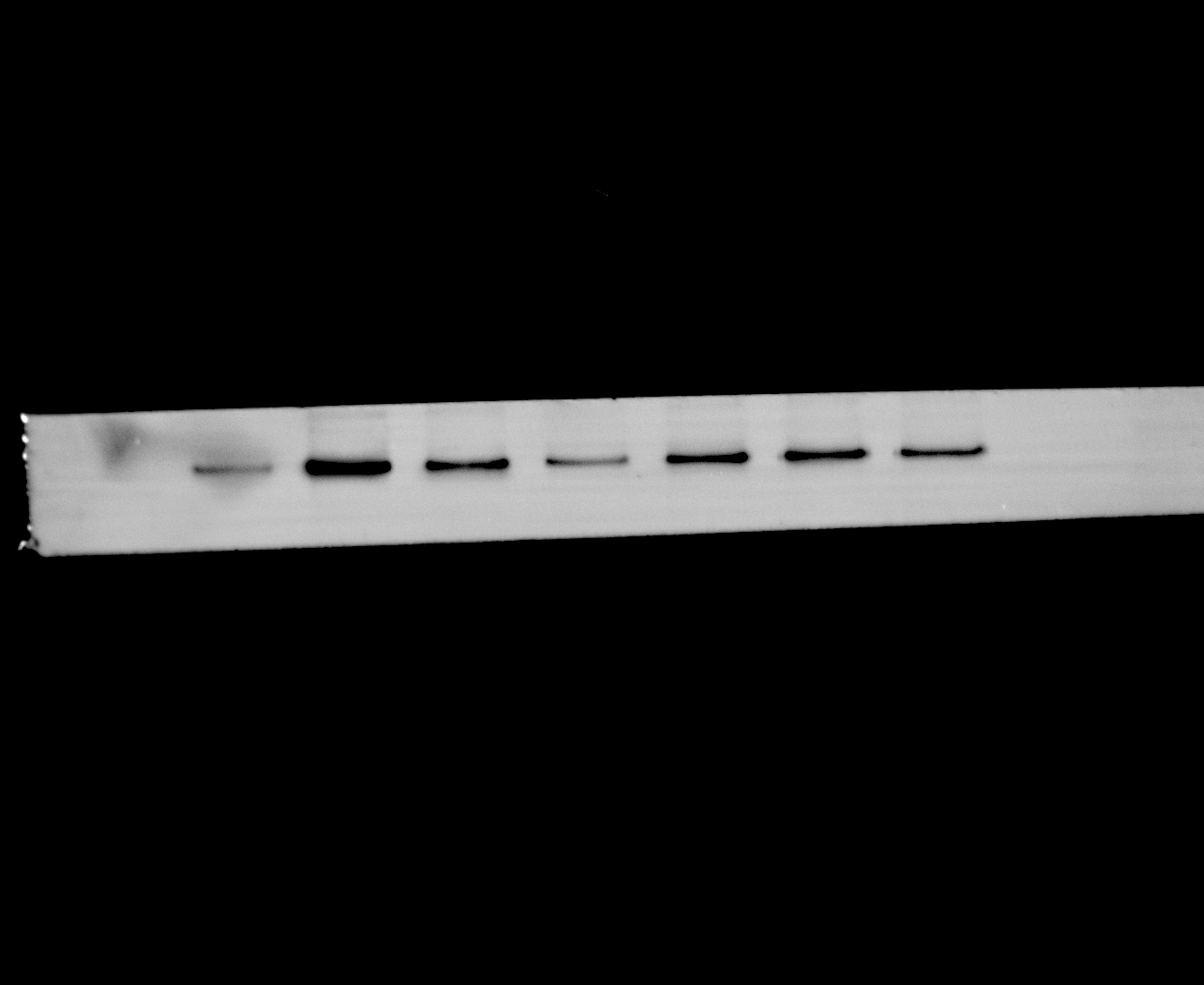

Supplement: Supplementary file 3 [file DataSheet9.ZIP › raw data/2.1.2/1.HMOX1.tif]

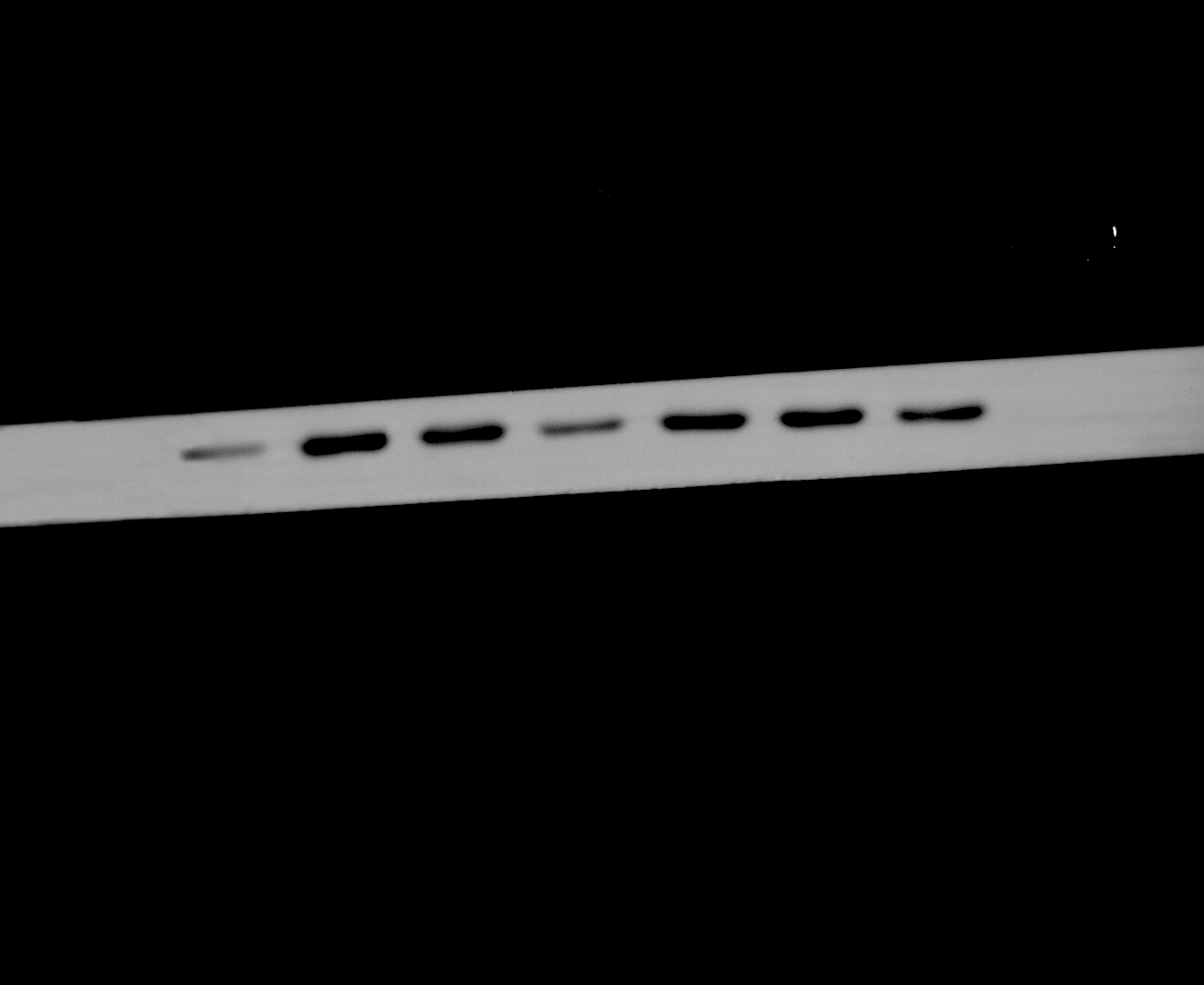

Supplement: Supplementary file 3 [file DataSheet9.ZIP › raw data/2.1.2/2.HIF-1a┴ (1).tif]

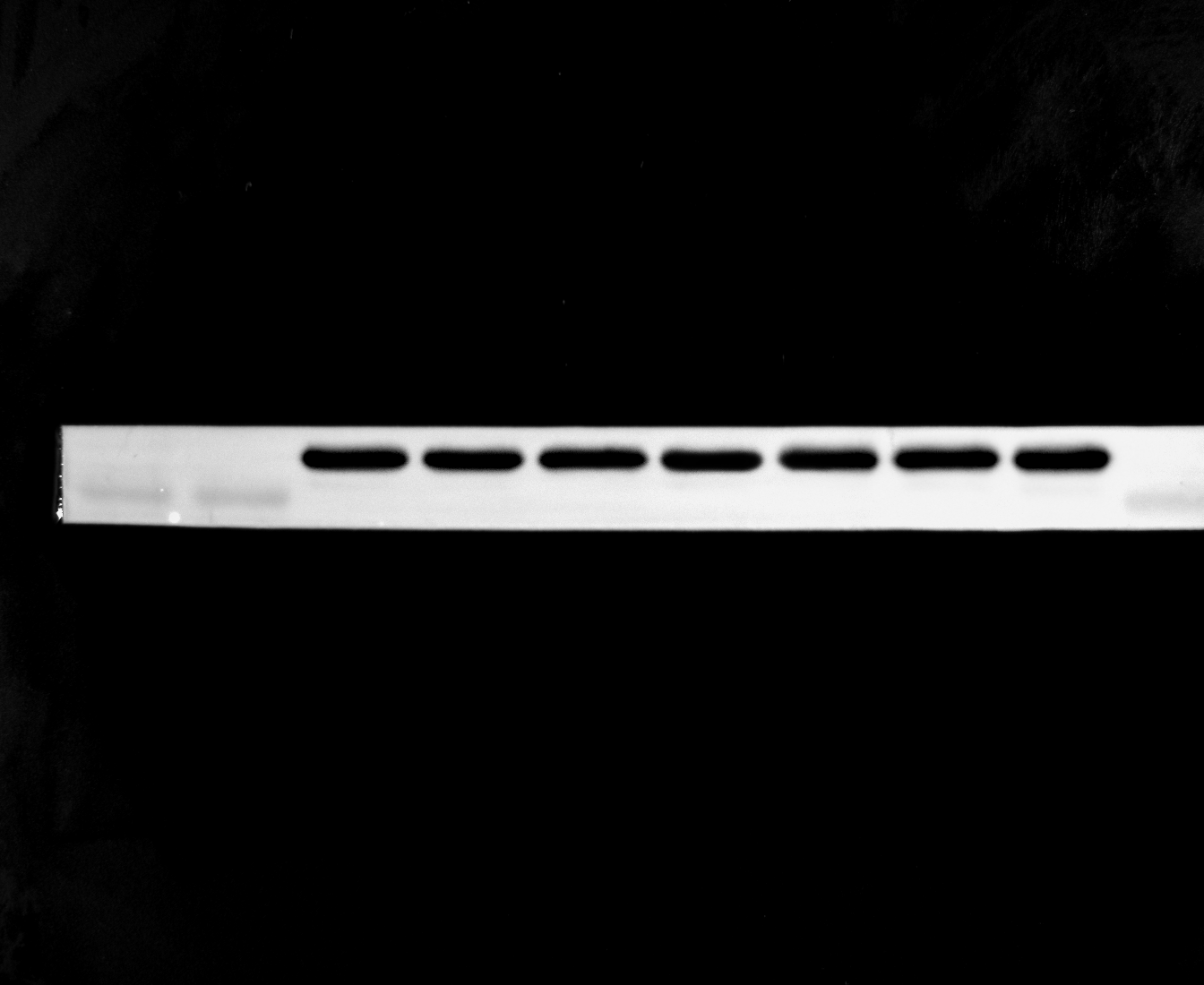

Supplement: Supplementary file 3 [file DataSheet9.ZIP › raw data/2.1.2/─┌▓╬ (1).tif]

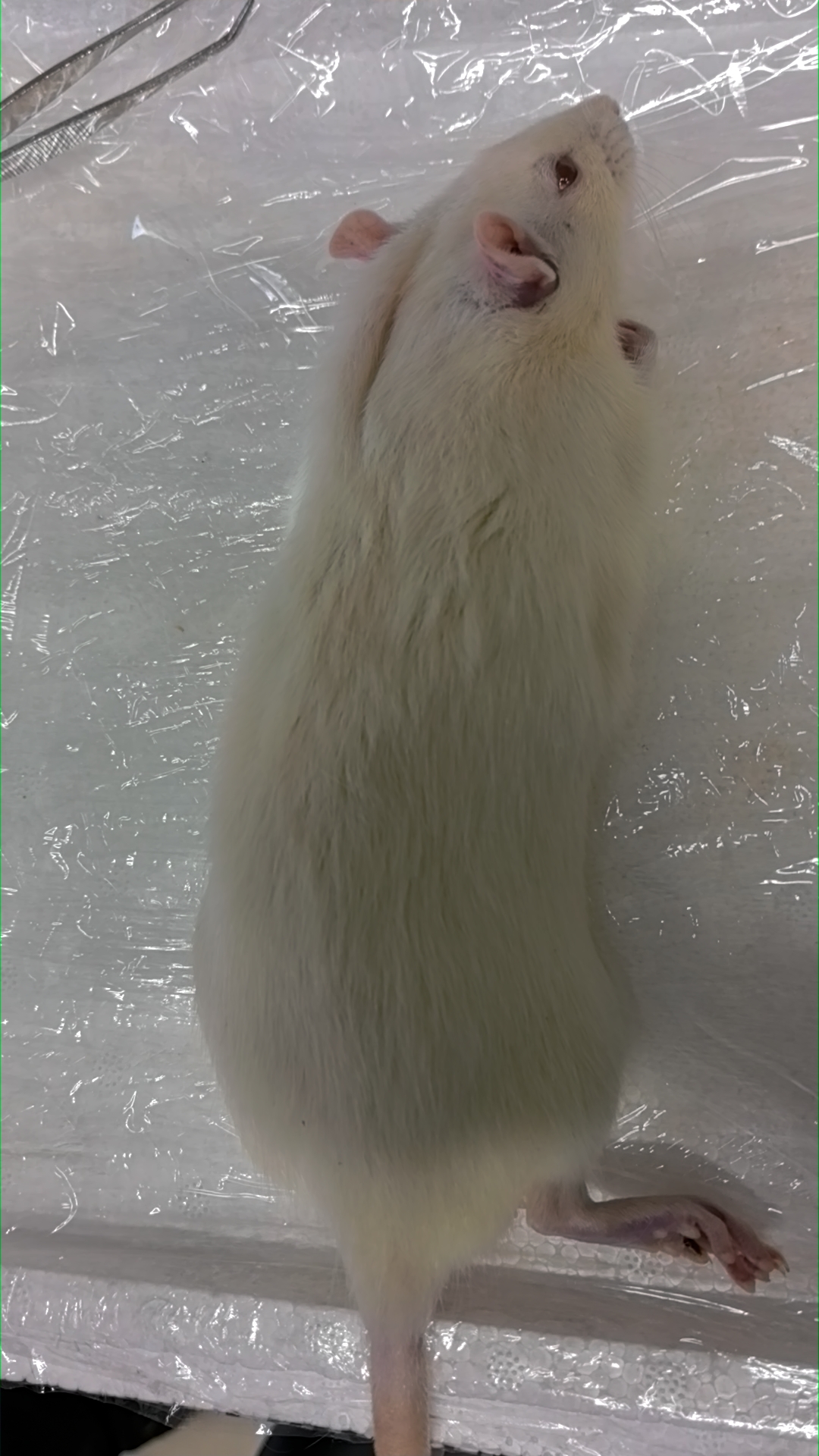

Supplement: Supplementary file 4 [file DataSheet4.ZIP › sodium fluorescein staining/1 (1).jpg]

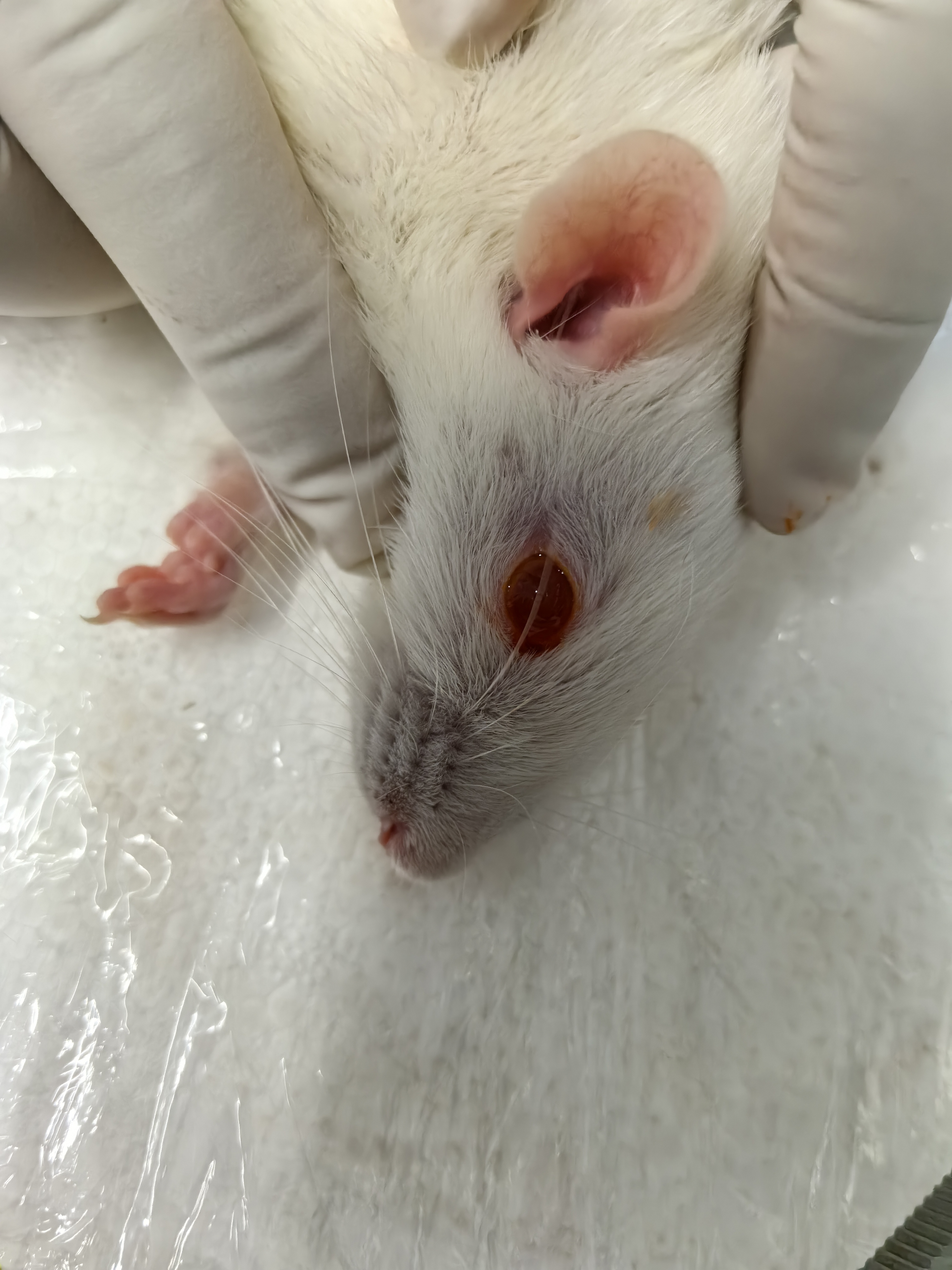

Supplement: Supplementary file 4 [file DataSheet4.ZIP › sodium fluorescein staining/1 (2).jpg]

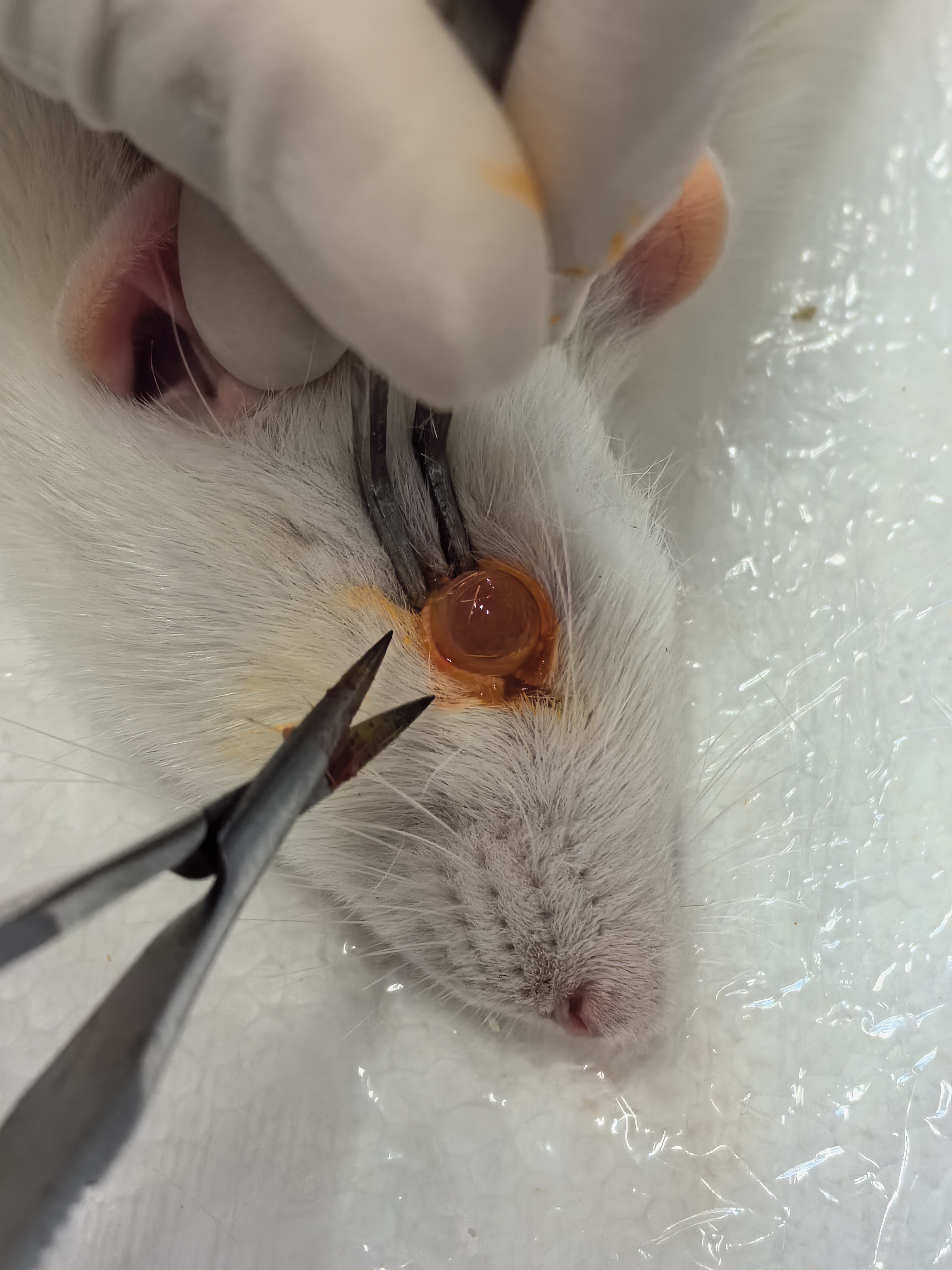

Supplement: Supplementary file 4 [file DataSheet4.ZIP › sodium fluorescein staining/1 (3).jpg]

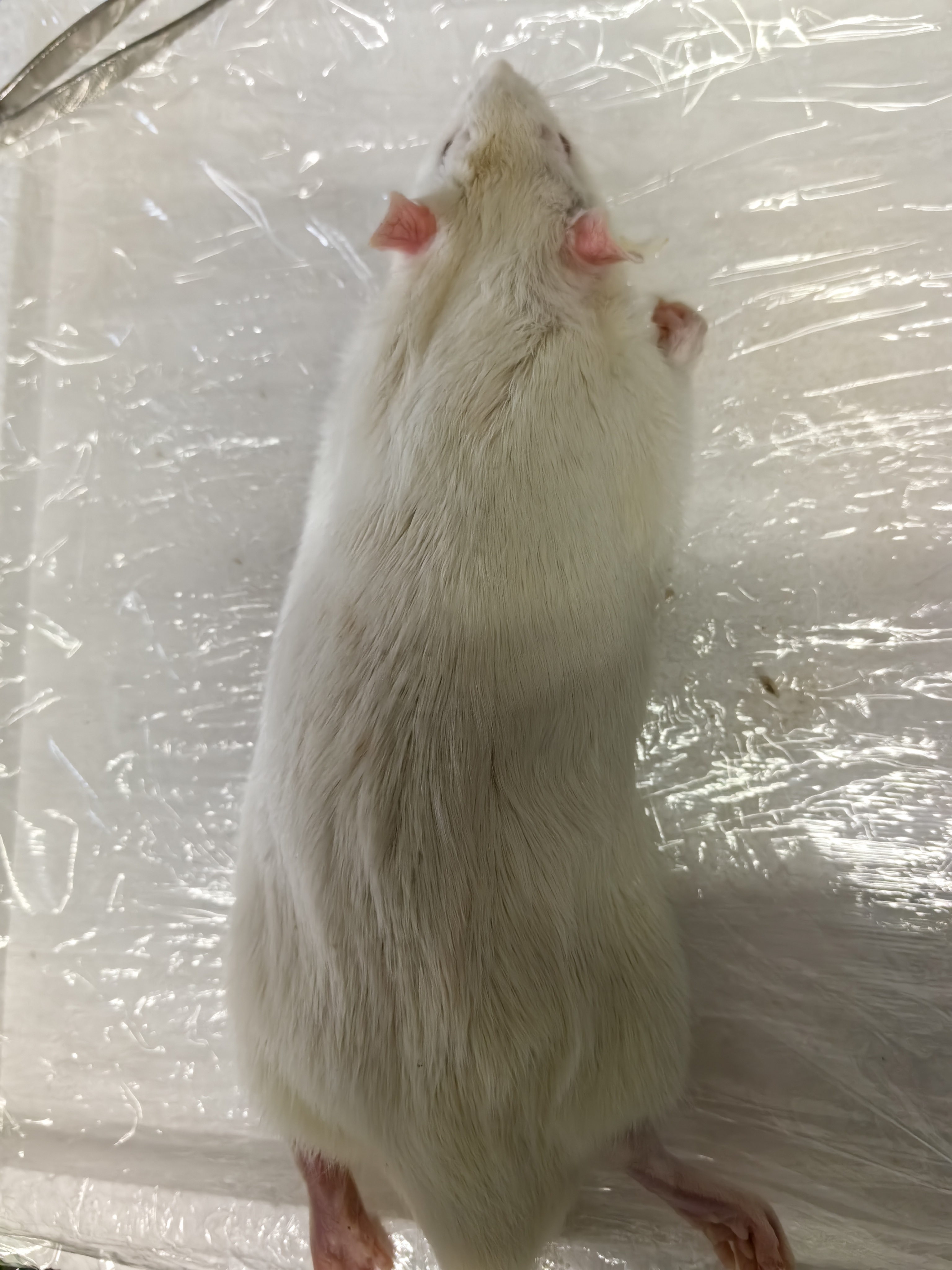

Supplement: Supplementary file 4 [file DataSheet4.ZIP › sodium fluorescein staining/1 (4).jpg]

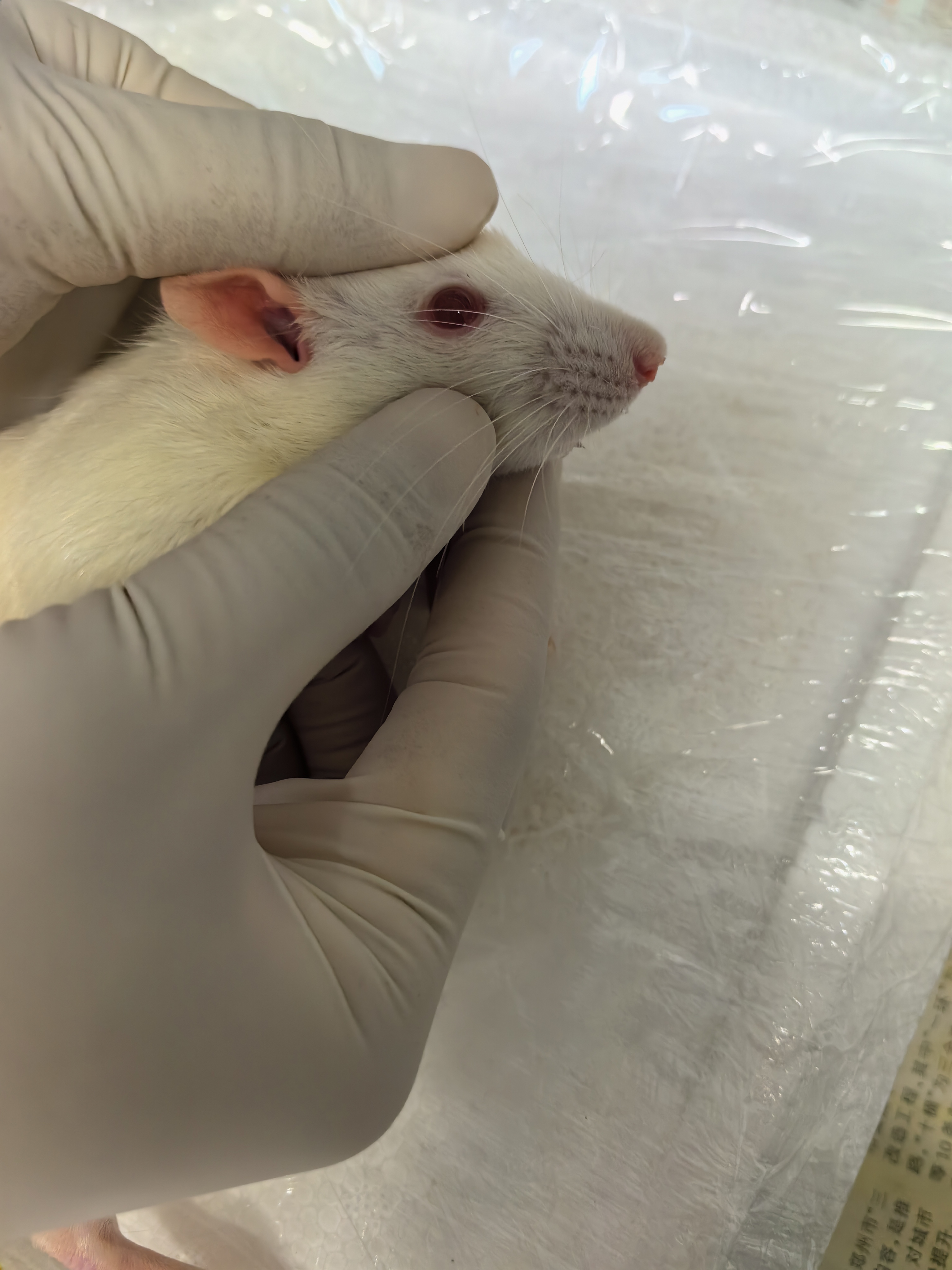

Supplement: Supplementary file 4 [file DataSheet4.ZIP › sodium fluorescein staining/1 (5).jpg]

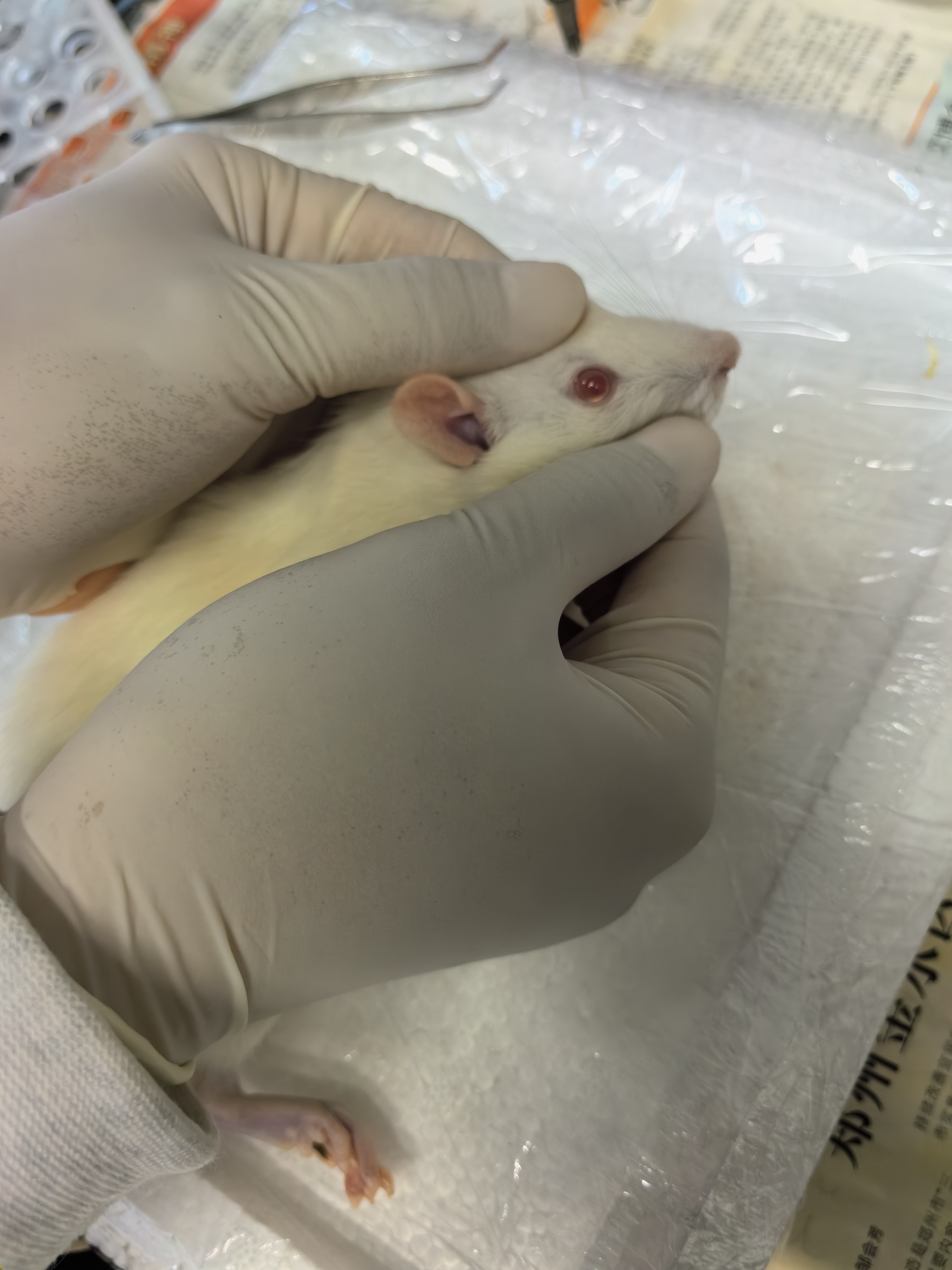

Supplement: Supplementary file 4 [file DataSheet4.ZIP › sodium fluorescein staining/1 (6).jpg]

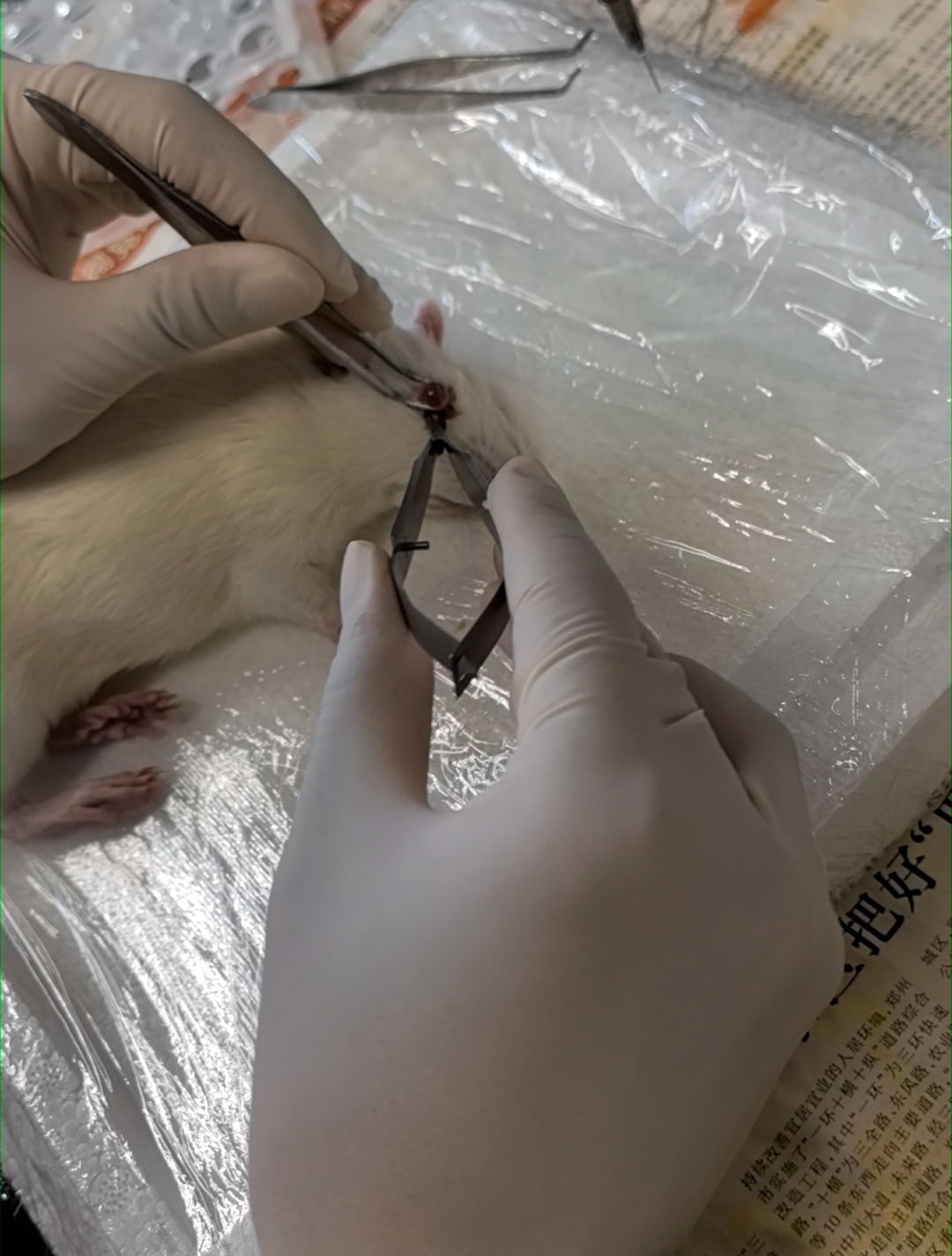

Supplement: Supplementary file 4 [file DataSheet4.ZIP › sodium fluorescein staining/1 (7).jpg]

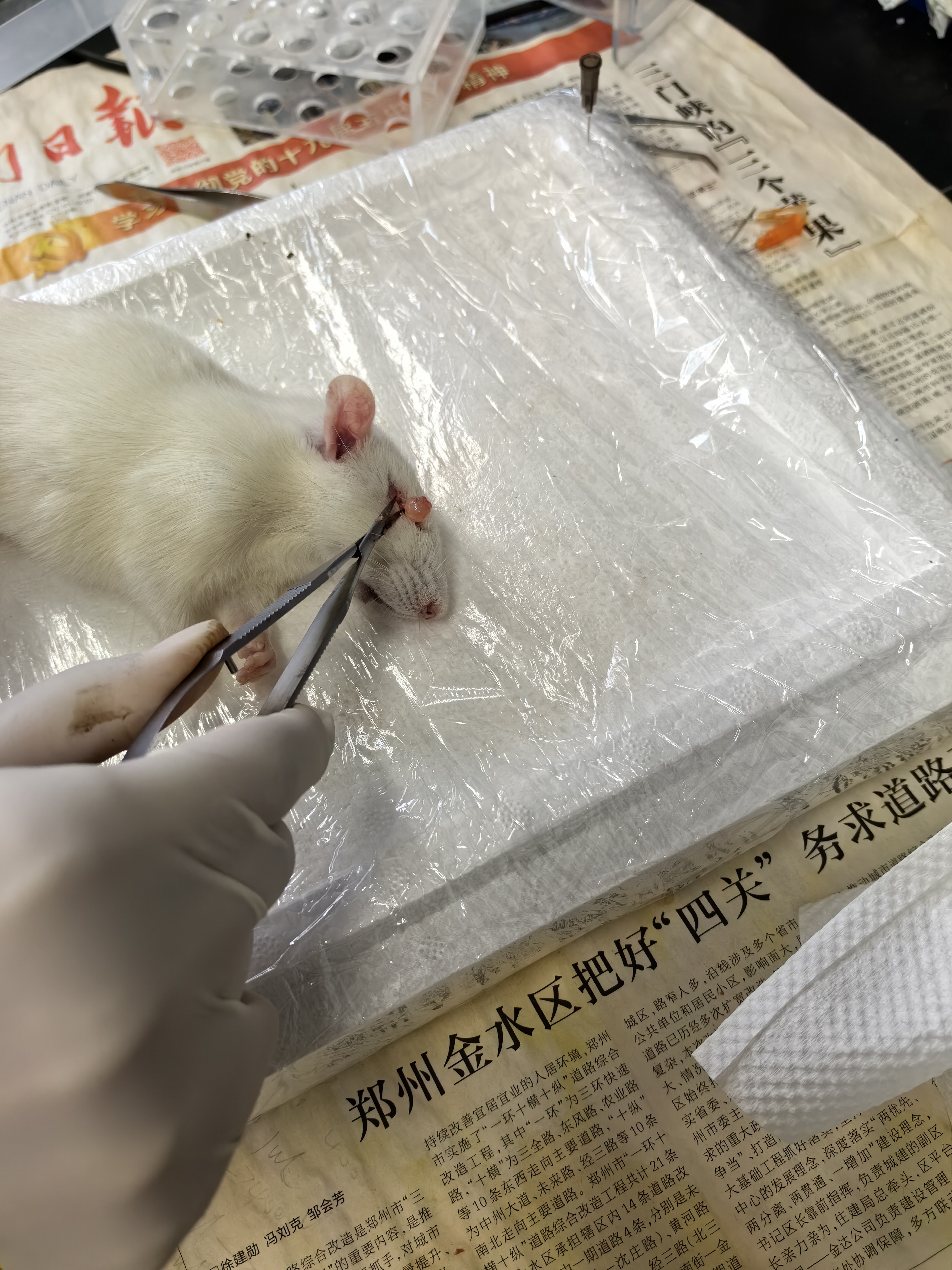

Supplement: Supplementary file 4 [file DataSheet4.ZIP › sodium fluorescein staining/sodium fluorescein staining-QXRM/1 (1).jpg]

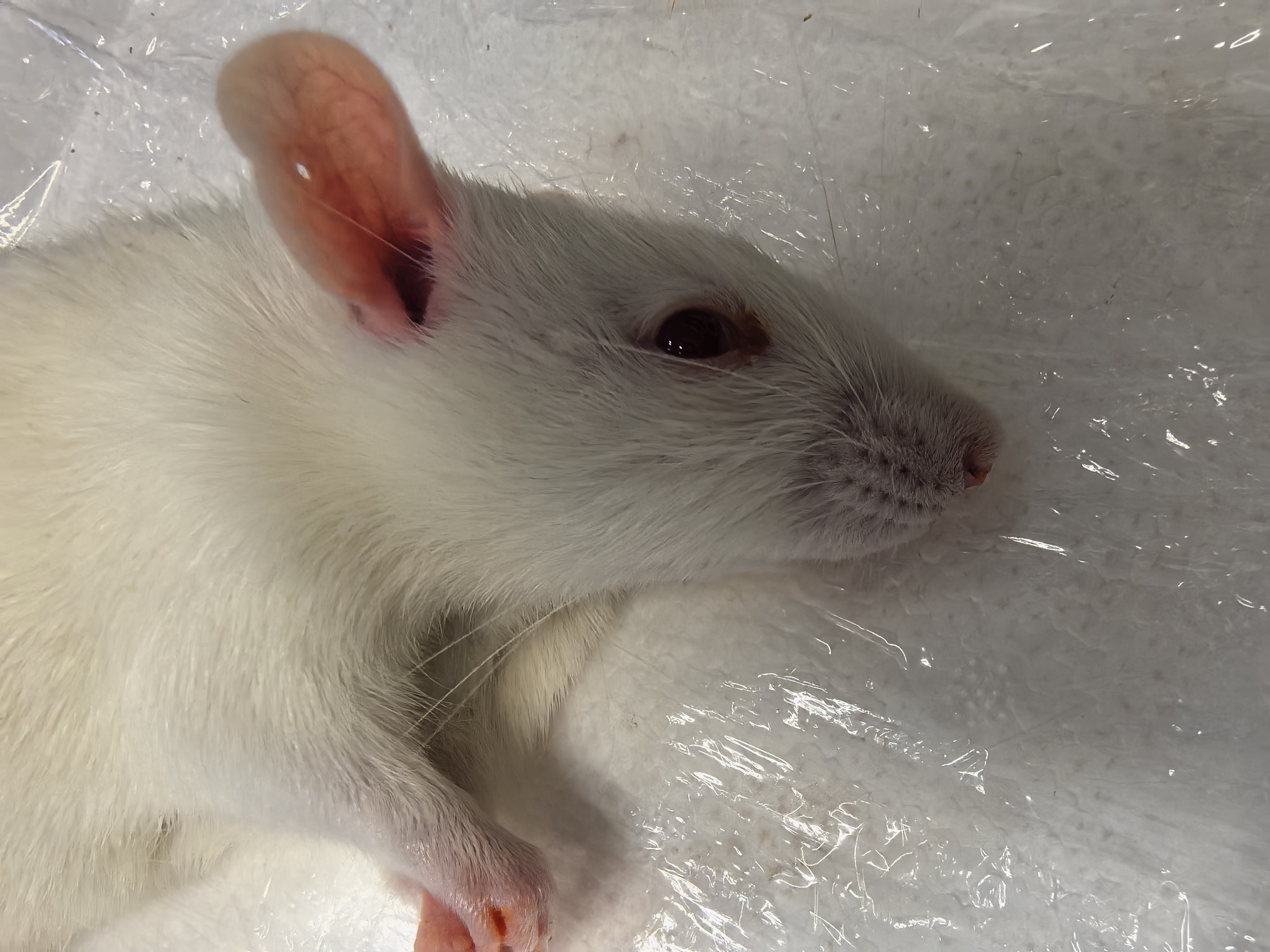

Supplement: Supplementary file 4 [file DataSheet4.ZIP › sodium fluorescein staining/sodium fluorescein staining-QXRM/1 (2).jpg]

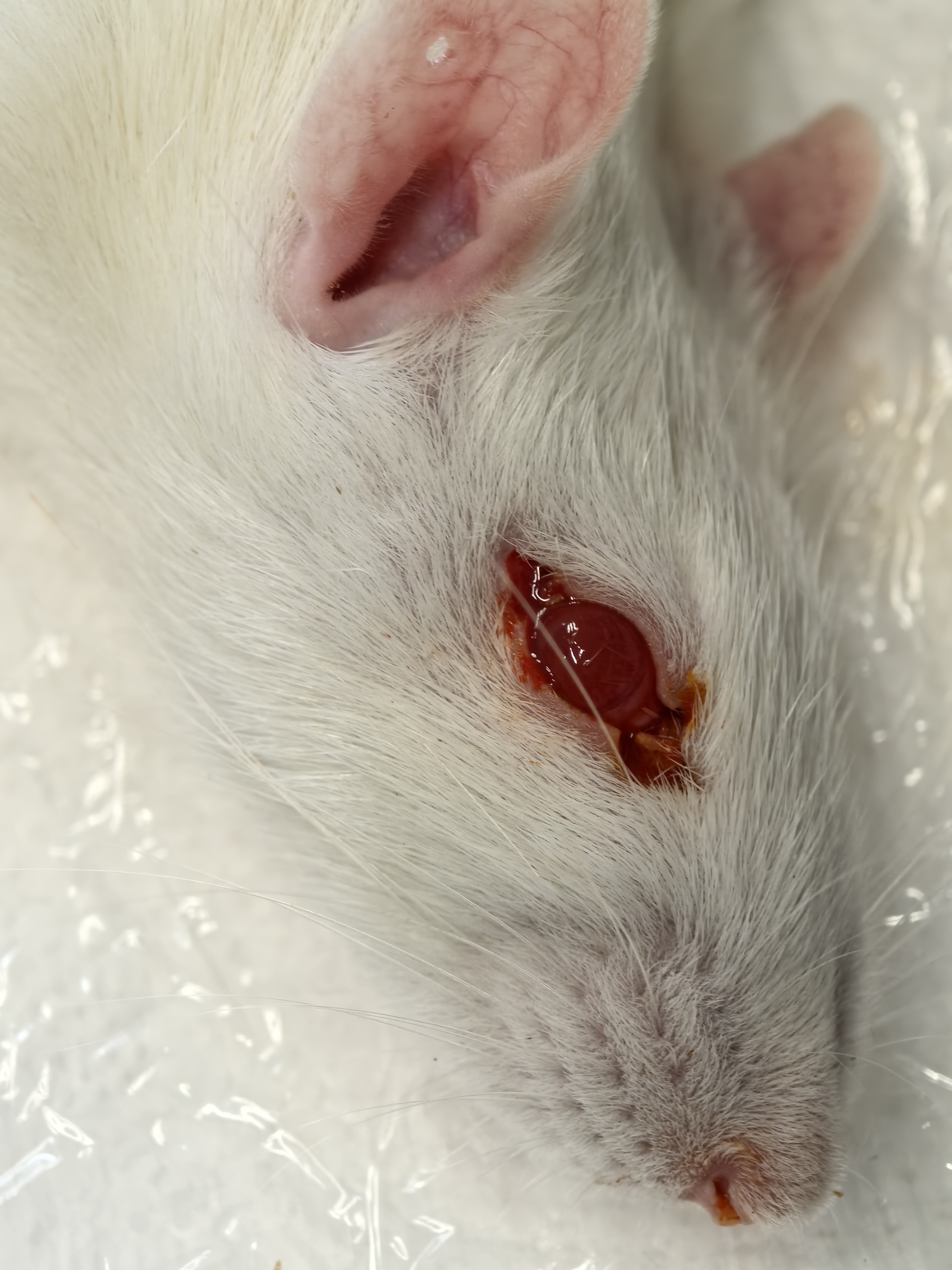

Supplement: Supplementary file 4 [file DataSheet4.ZIP › sodium fluorescein staining/sodium fluorescein staining-QXRM/1 (3).jpg]

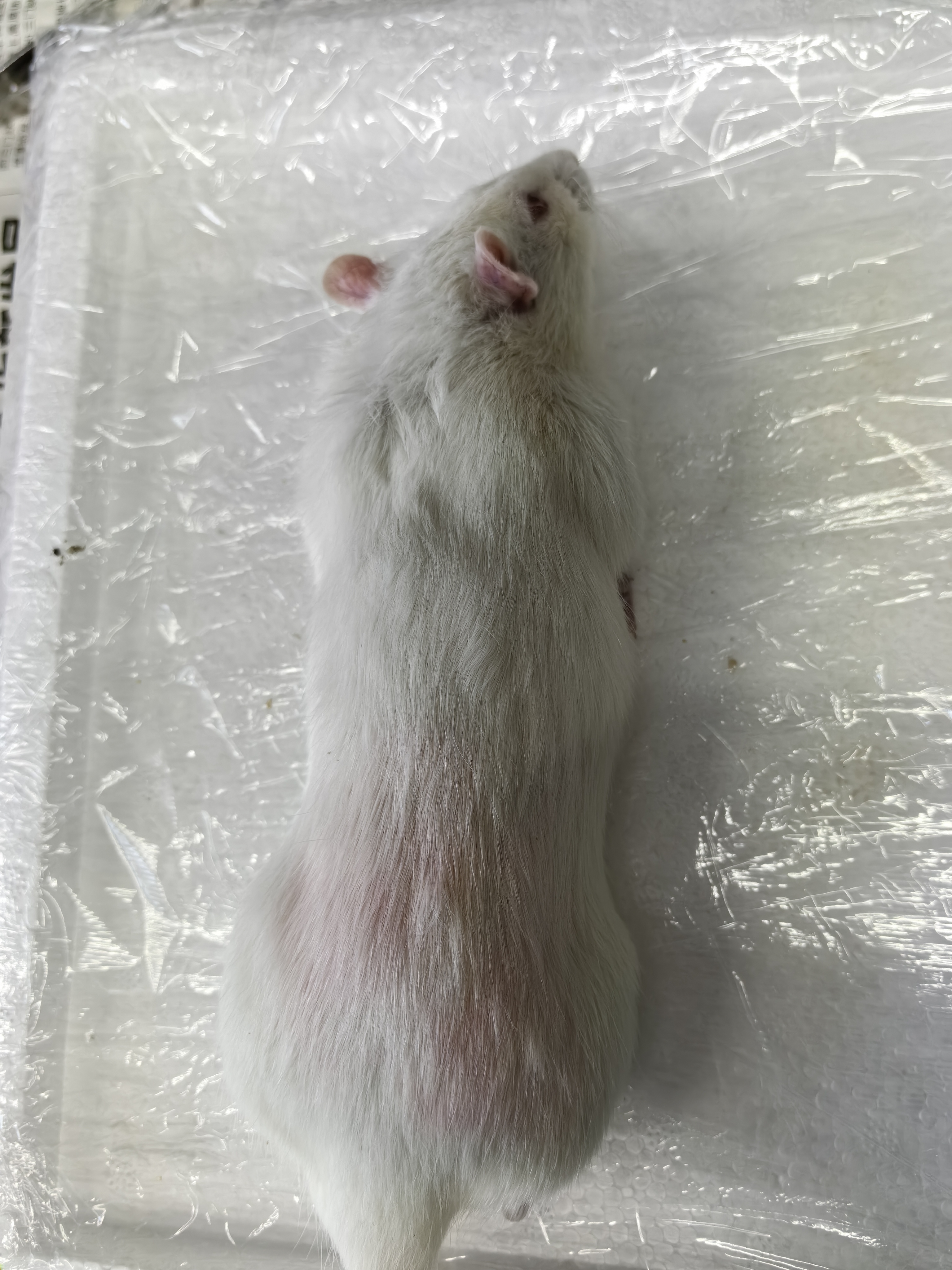

Supplement: Supplementary file 4 [file DataSheet4.ZIP › sodium fluorescein staining/sodium fluorescein staining-QXRM/1 (4).jpg]

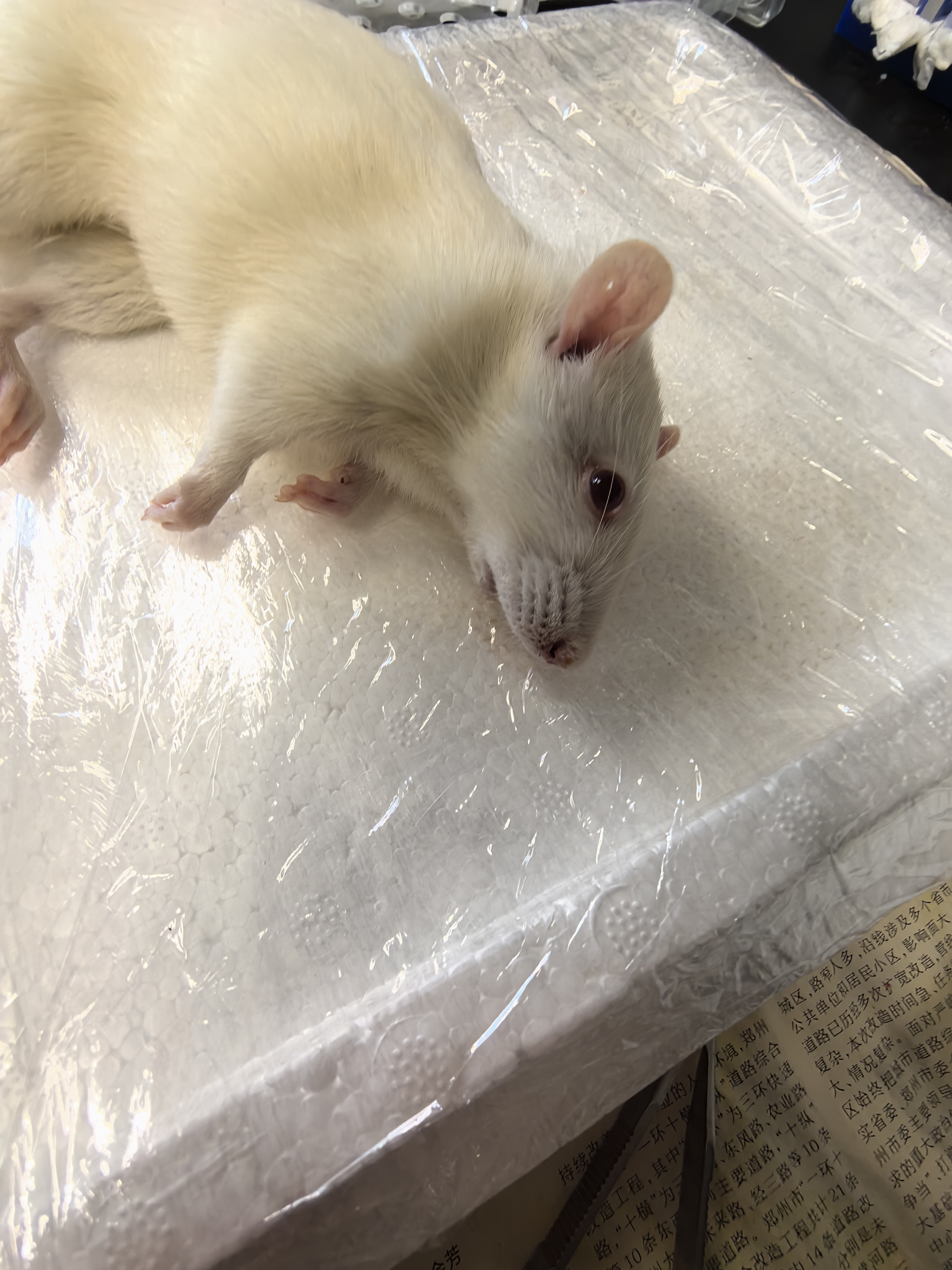

Supplement: Supplementary file 4 [file DataSheet4.ZIP › sodium fluorescein staining/sodium fluorescein staining-QXRM/1 (5).jpg]

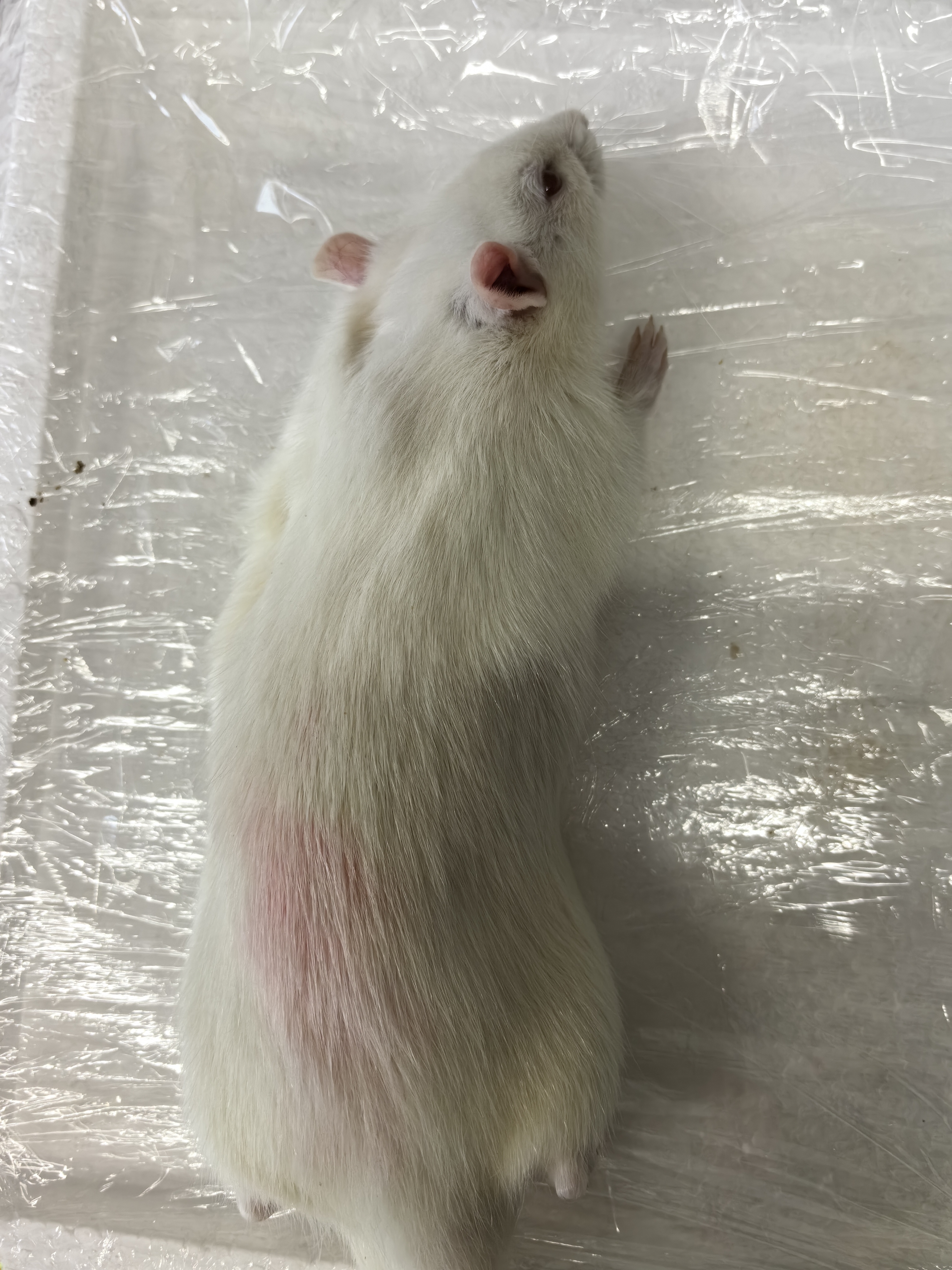

Supplement: Supplementary file 4 [file DataSheet4.ZIP › sodium fluorescein staining/sodium fluorescein staining-QXRM/1 (6).jpg]

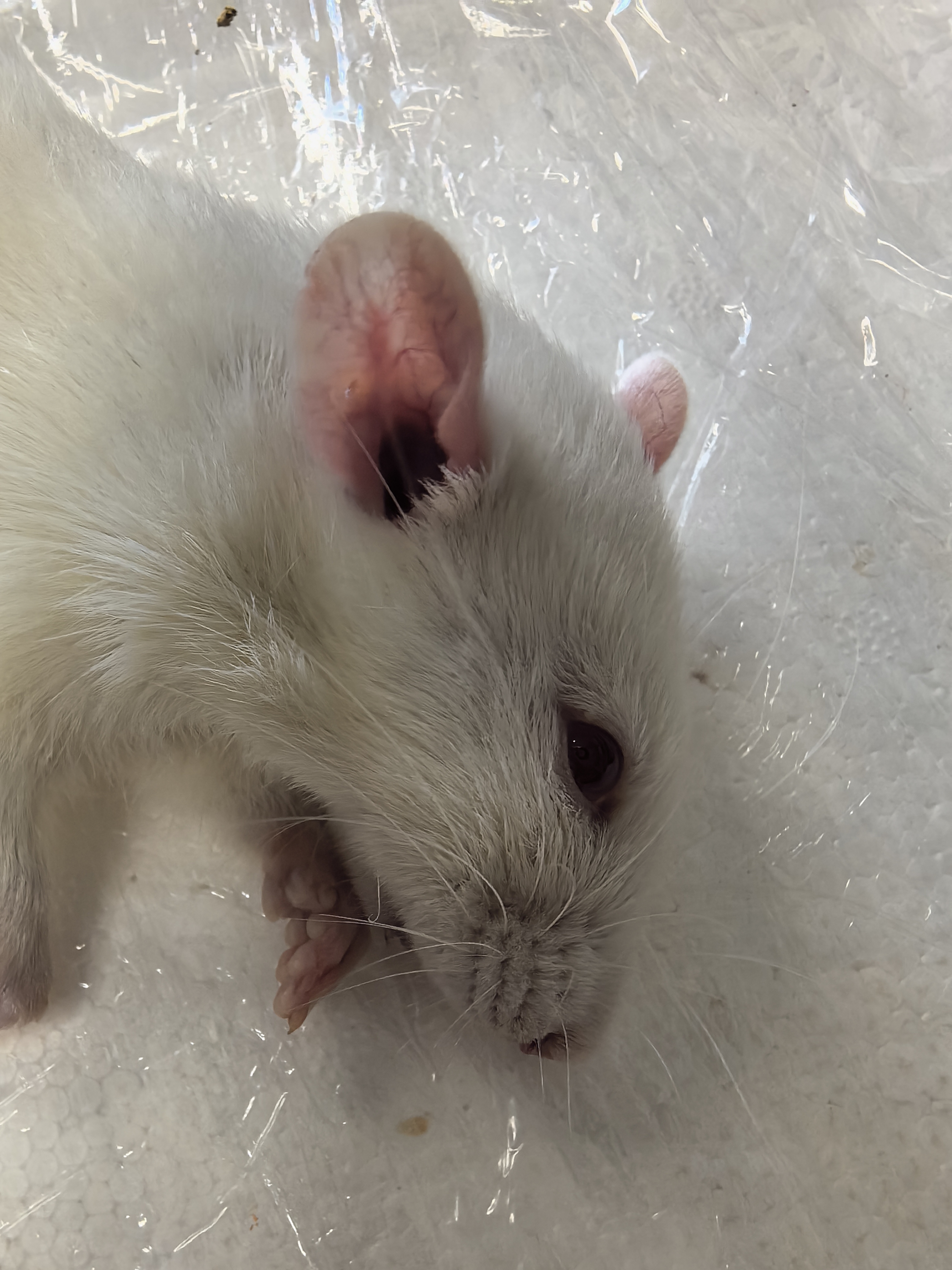

Supplement: Supplementary file 4 [file DataSheet4.ZIP › sodium fluorescein staining/sodium fluorescein staining-QXRM/1 (7).jpg]

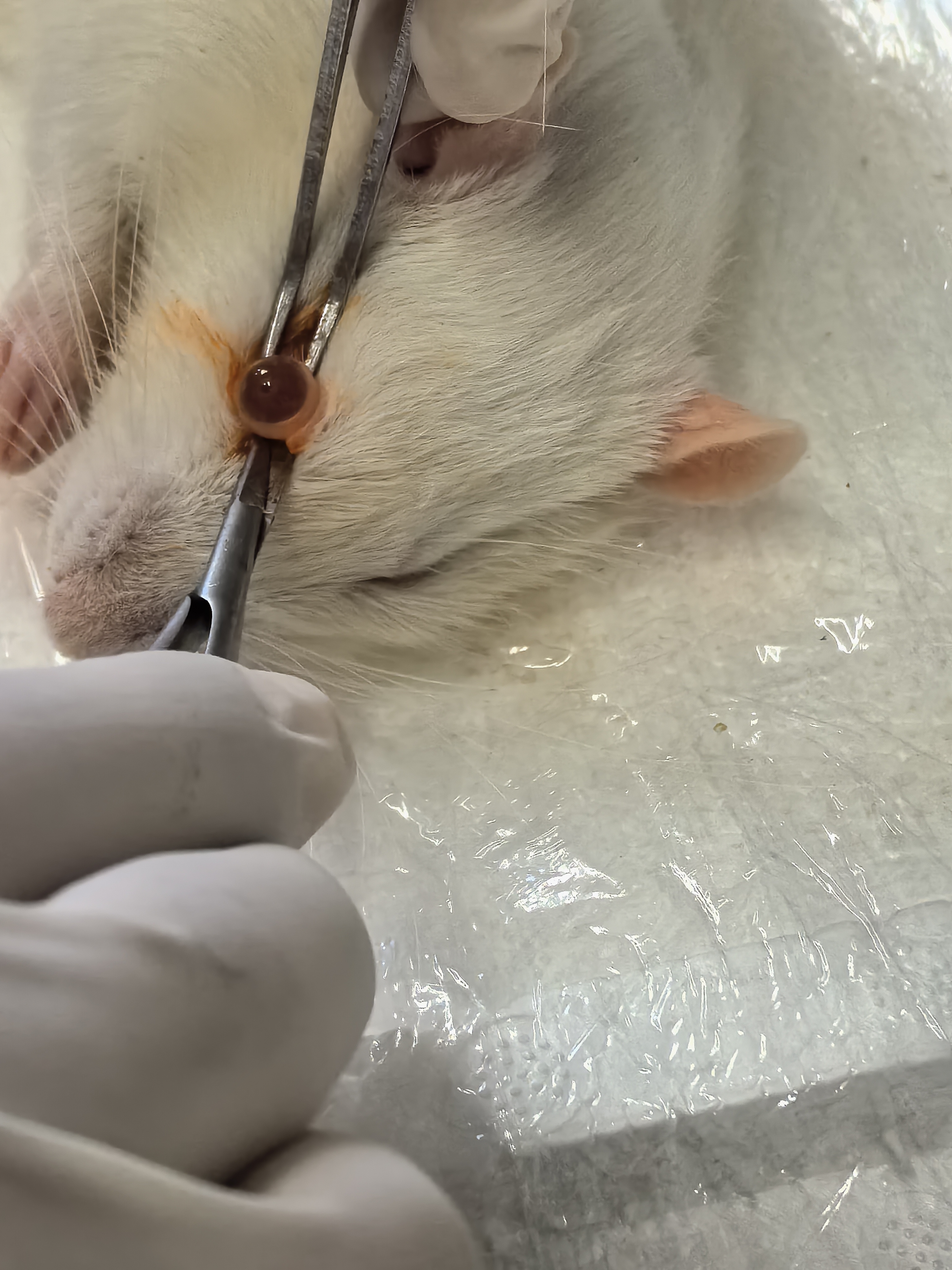

Supplement: Supplementary file 7 [file DataSheet2.ZIP › contr/1 (1).jpg]

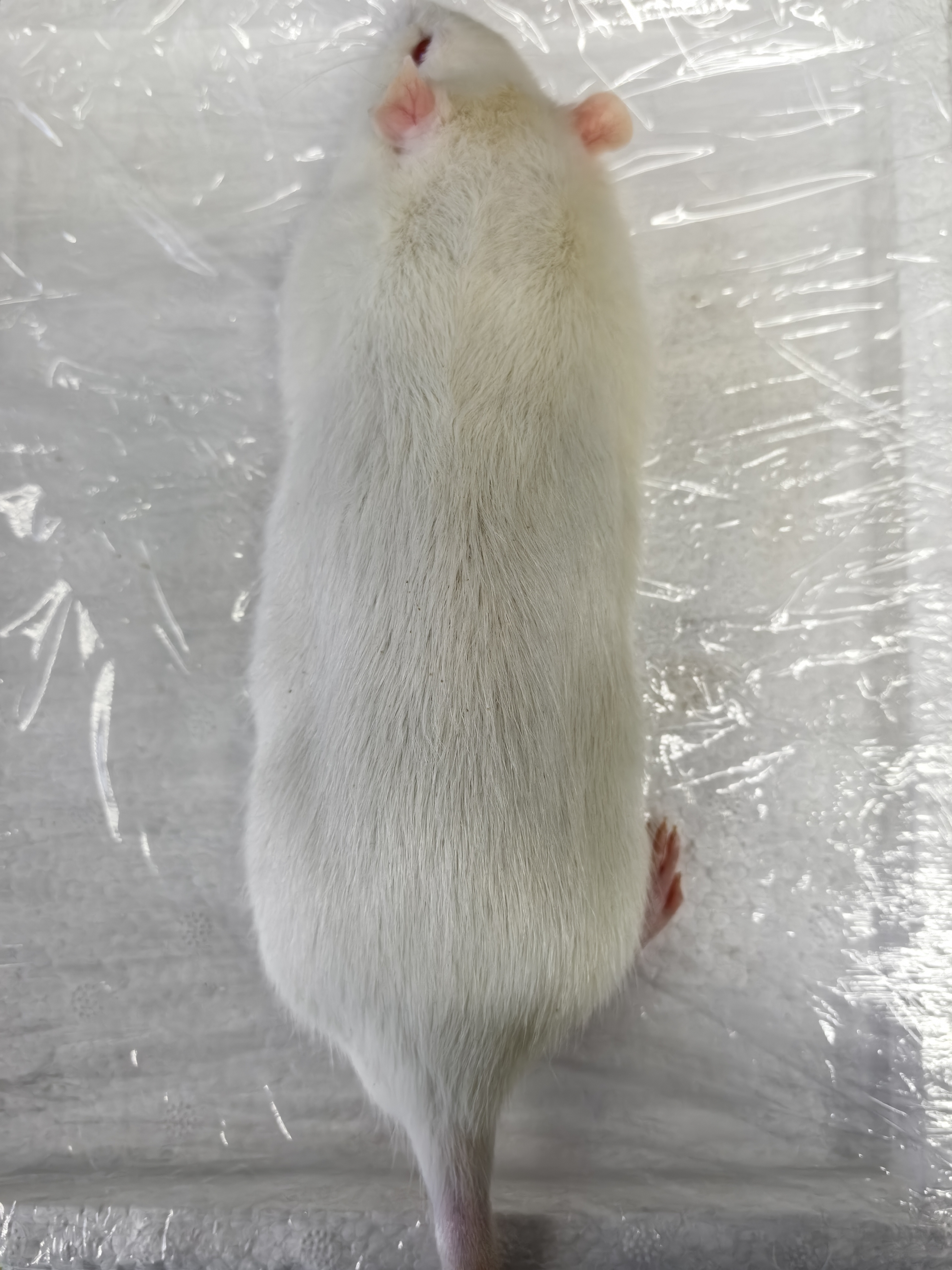

Supplement: Supplementary file 7 [file DataSheet2.ZIP › contr/1 (2).jpg]

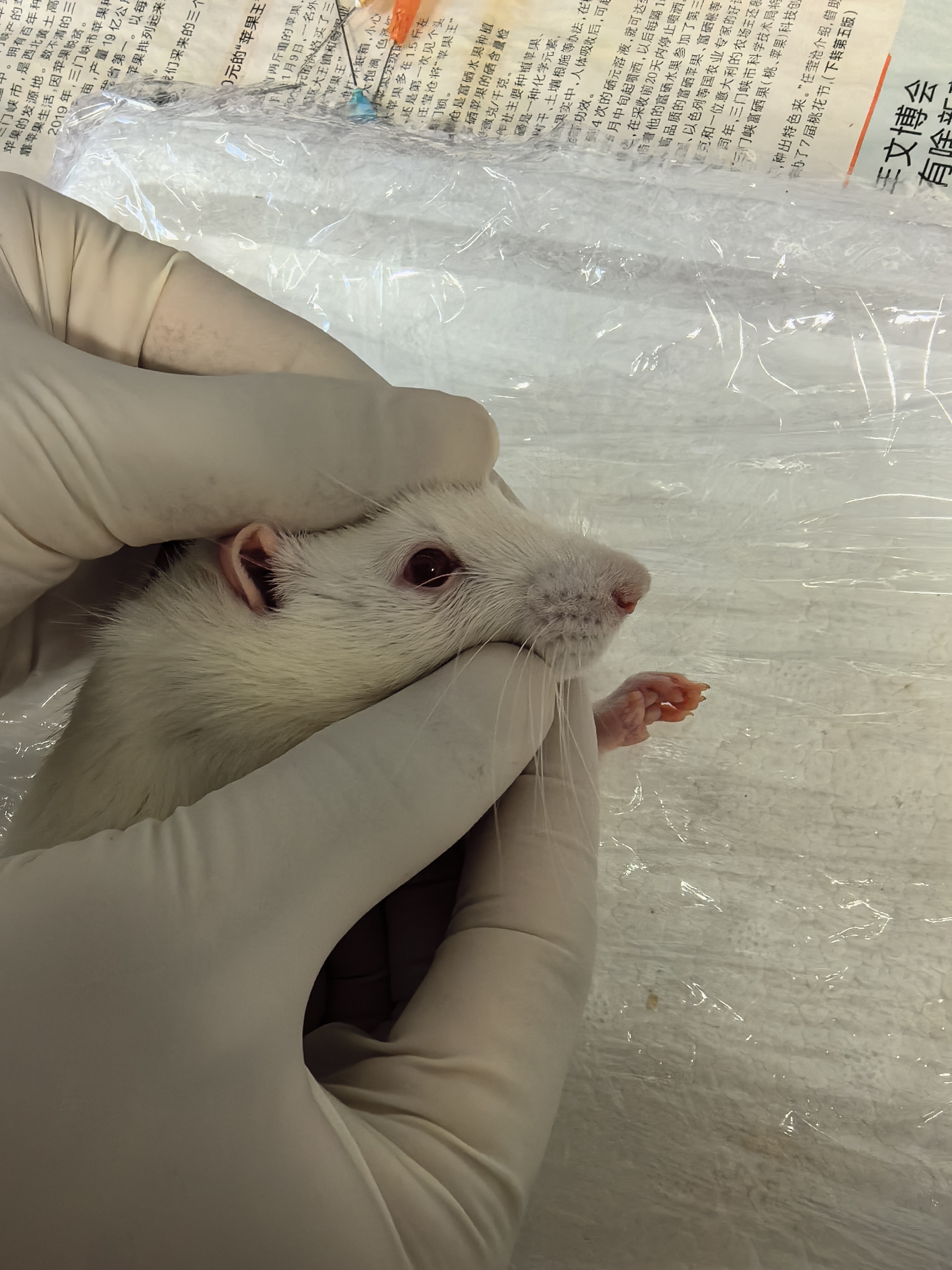

Supplement: Supplementary file 7 [file DataSheet2.ZIP › contr/1 (3).jpg]

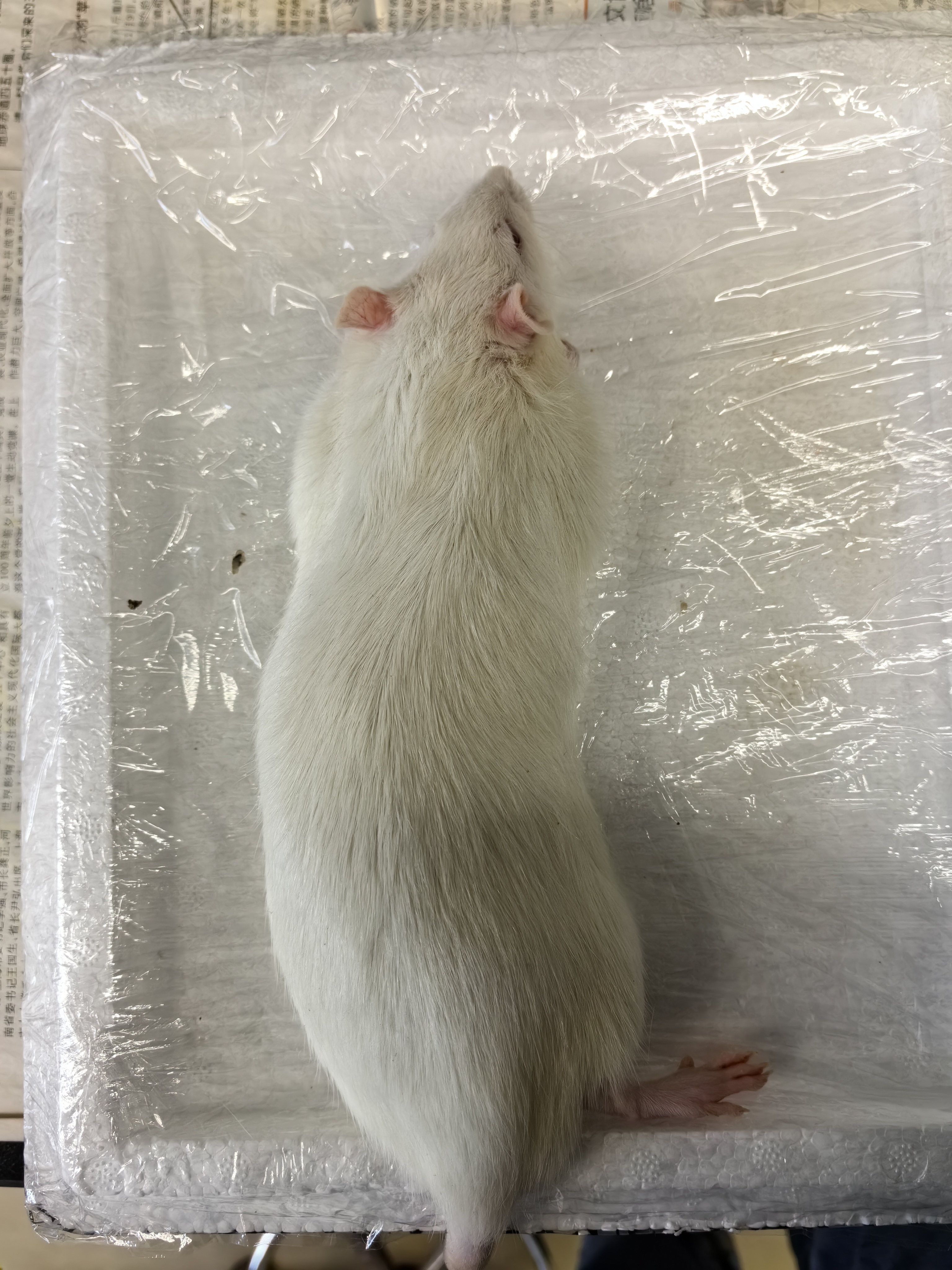

Supplement: Supplementary file 7 [file DataSheet2.ZIP › contr/1 (4).jpg]

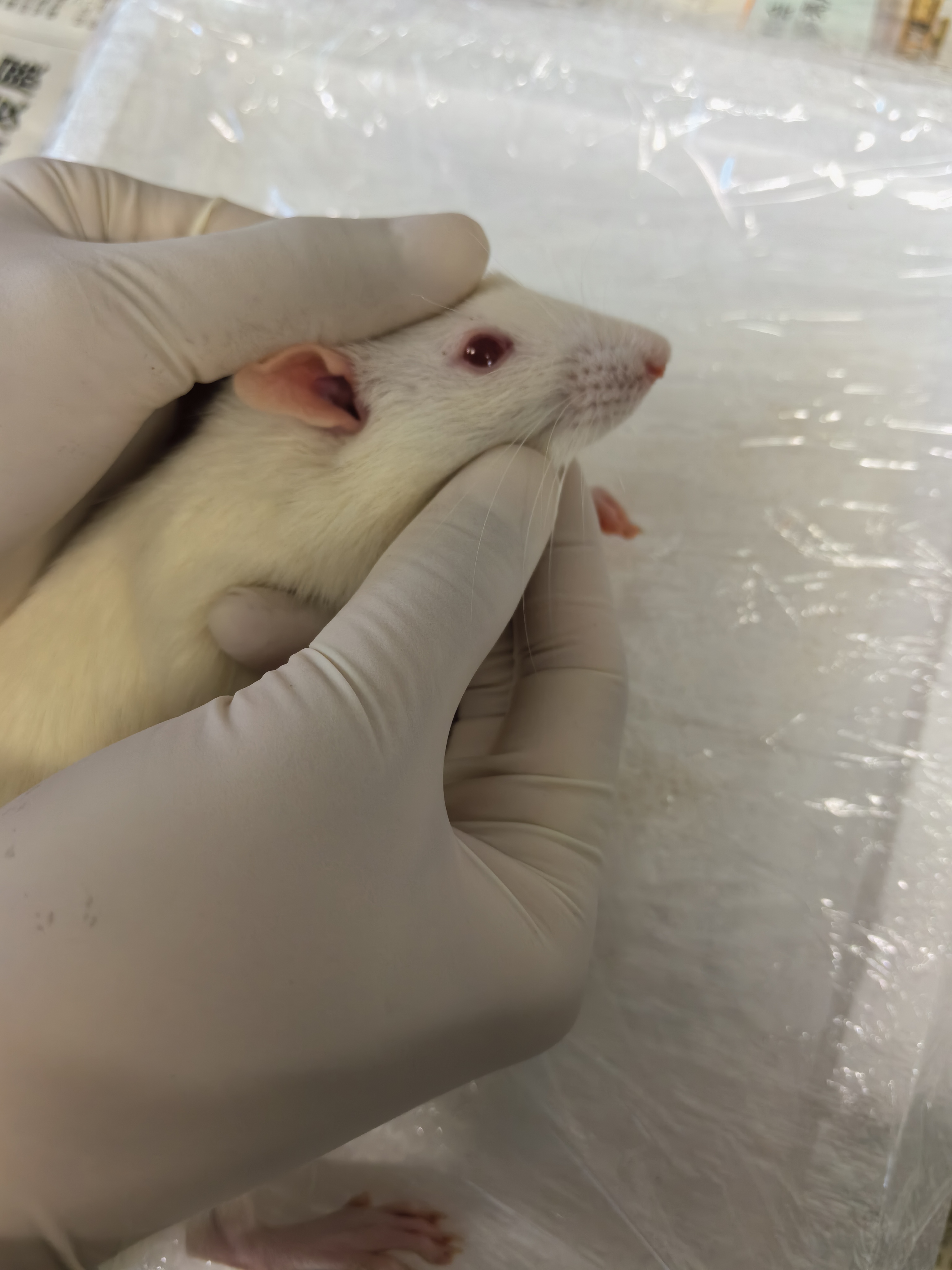

Supplement: Supplementary file 7 [file DataSheet2.ZIP › contr/1 (5).jpg]

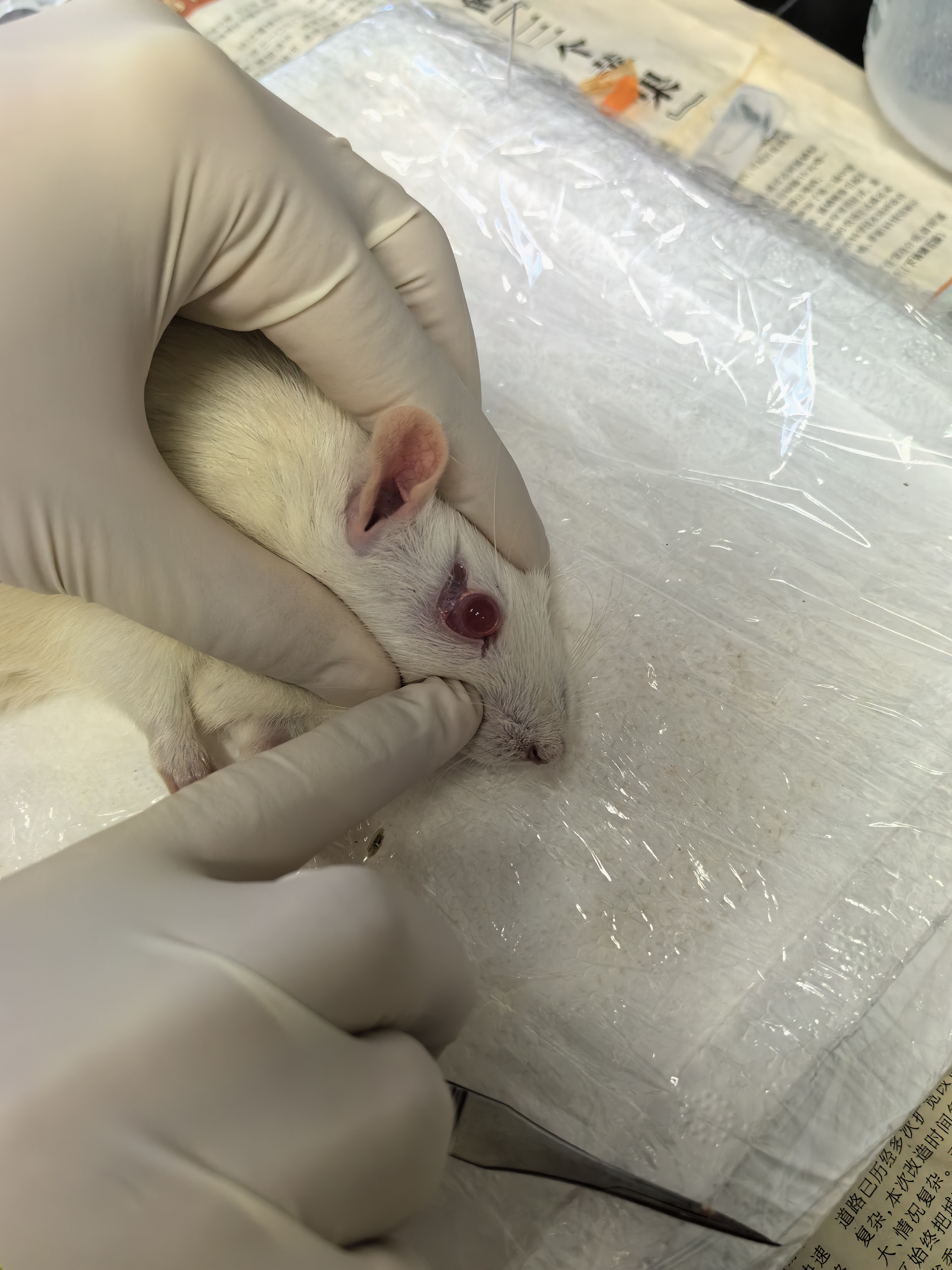

Supplement: Supplementary file 7 [file DataSheet2.ZIP › contr/1 (6).jpg]

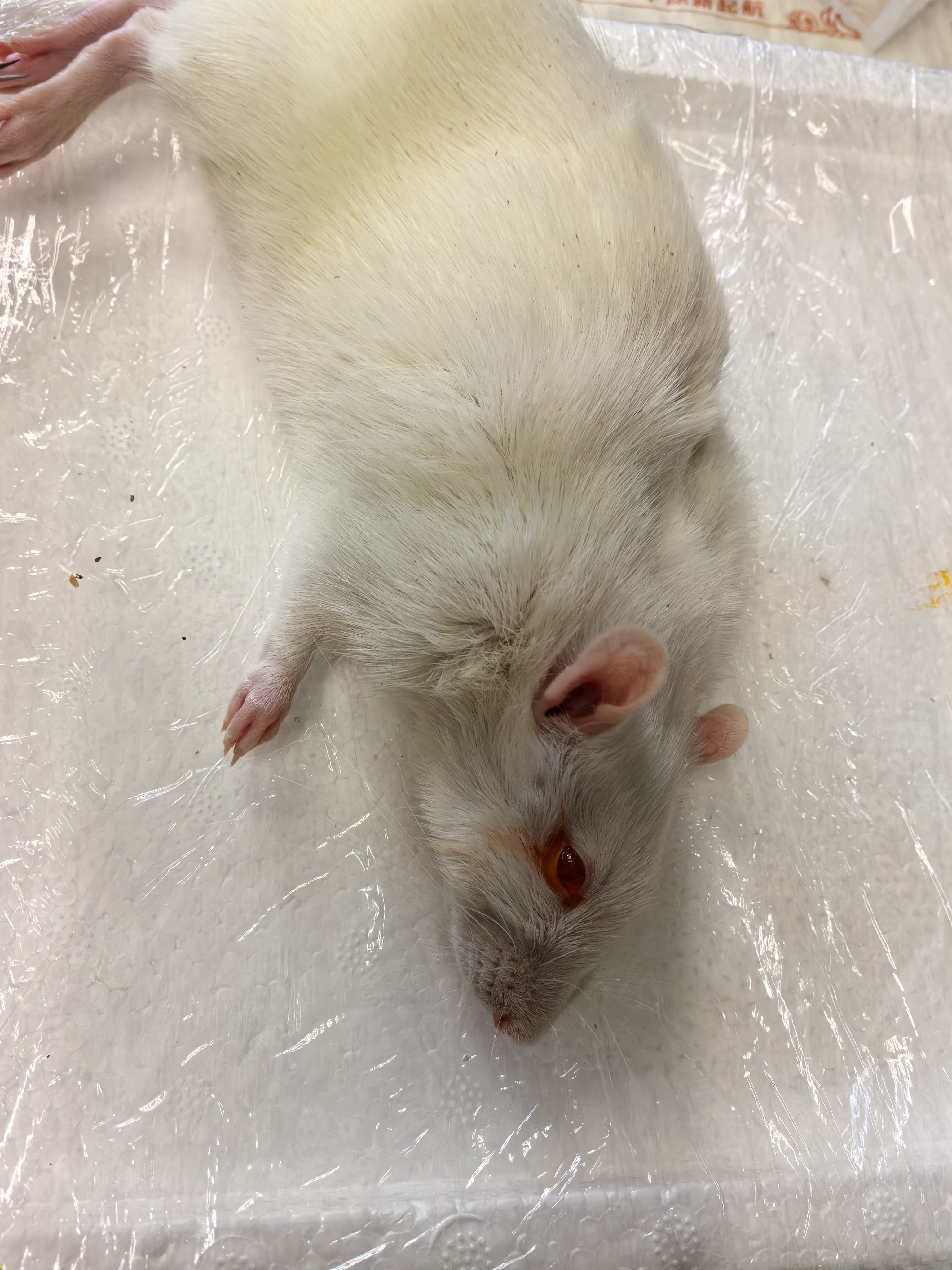

Supplement: Supplementary file 7 [file DataSheet2.ZIP › contr/1 (7).jpg]

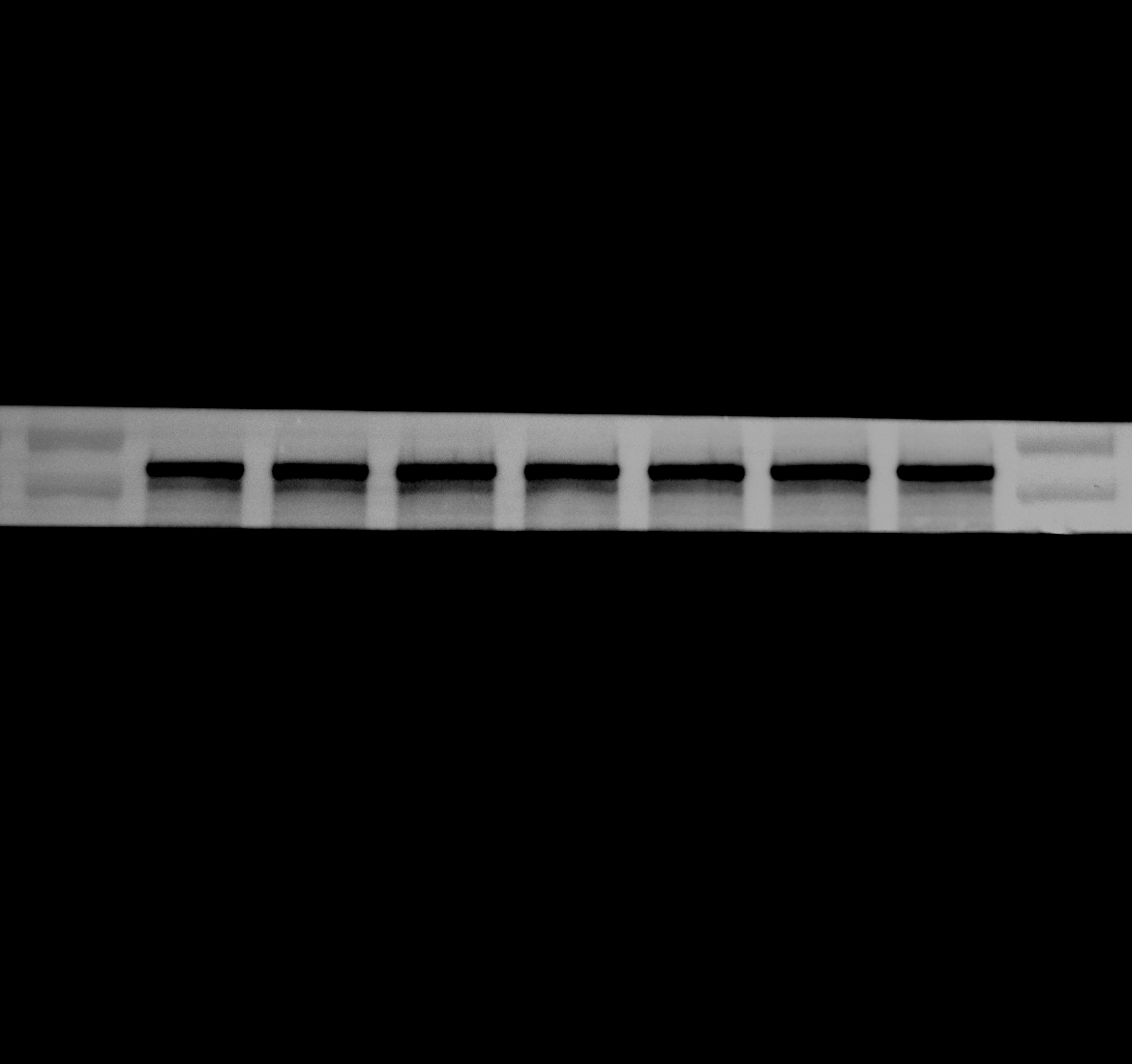

Supplement: Supplementary file 10 [file DataSheet7.ZIP › WB/1/gapdh.tif]

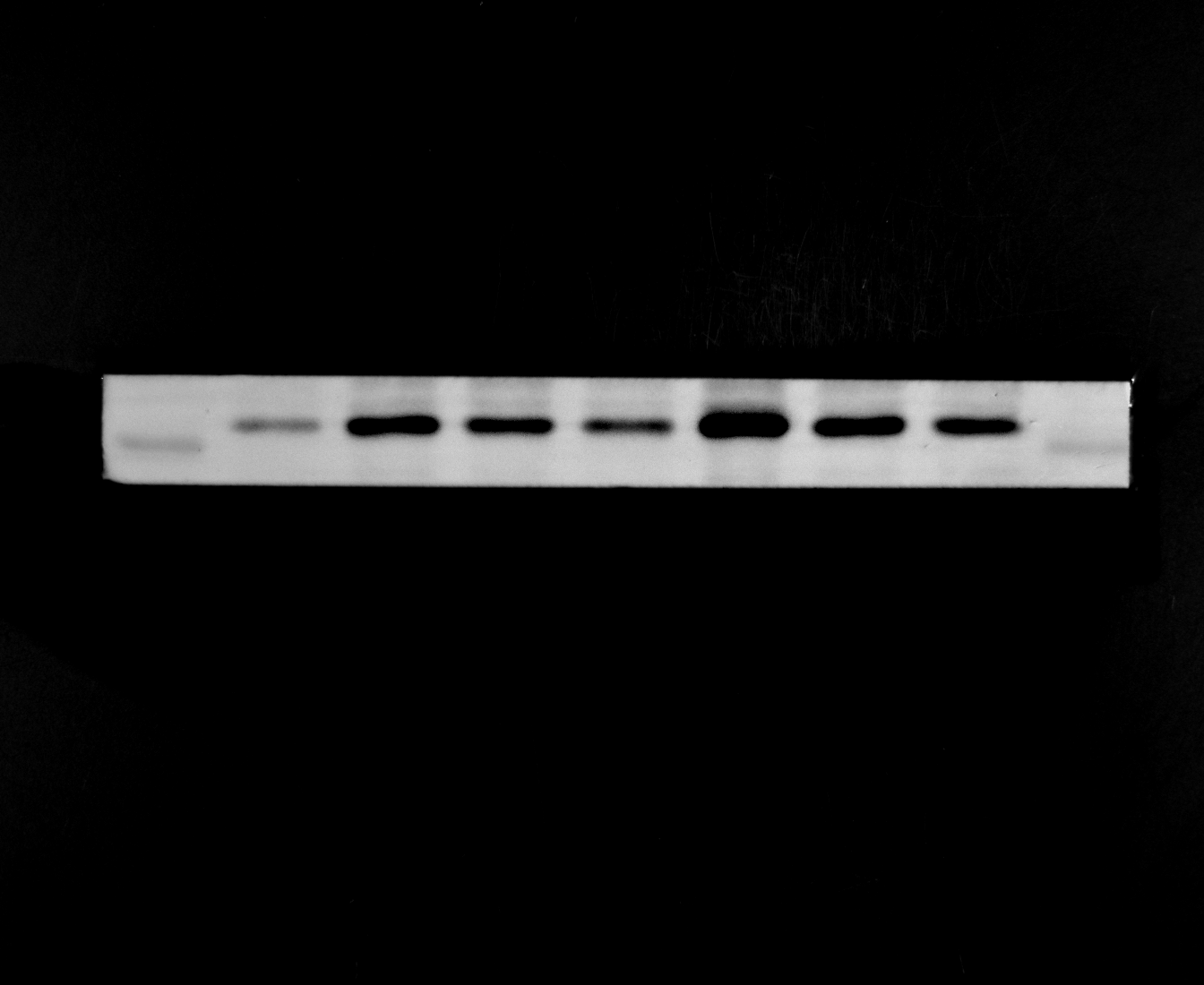

Supplement: Supplementary file 10 [file DataSheet7.ZIP › WB/1/HIF-1a┴.tif]

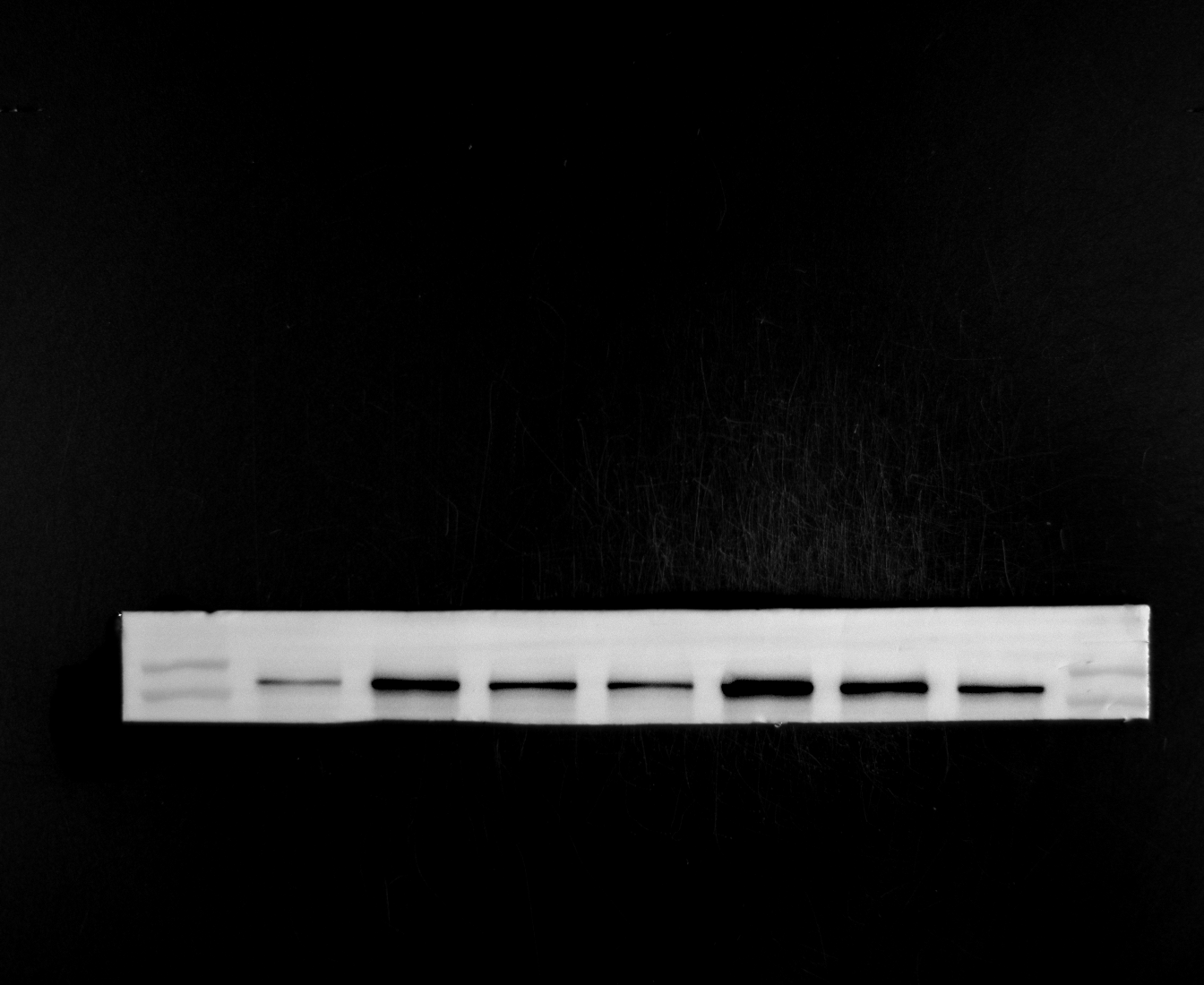

Supplement: Supplementary file 10 [file DataSheet7.ZIP › WB/1/HMOX1.tif]

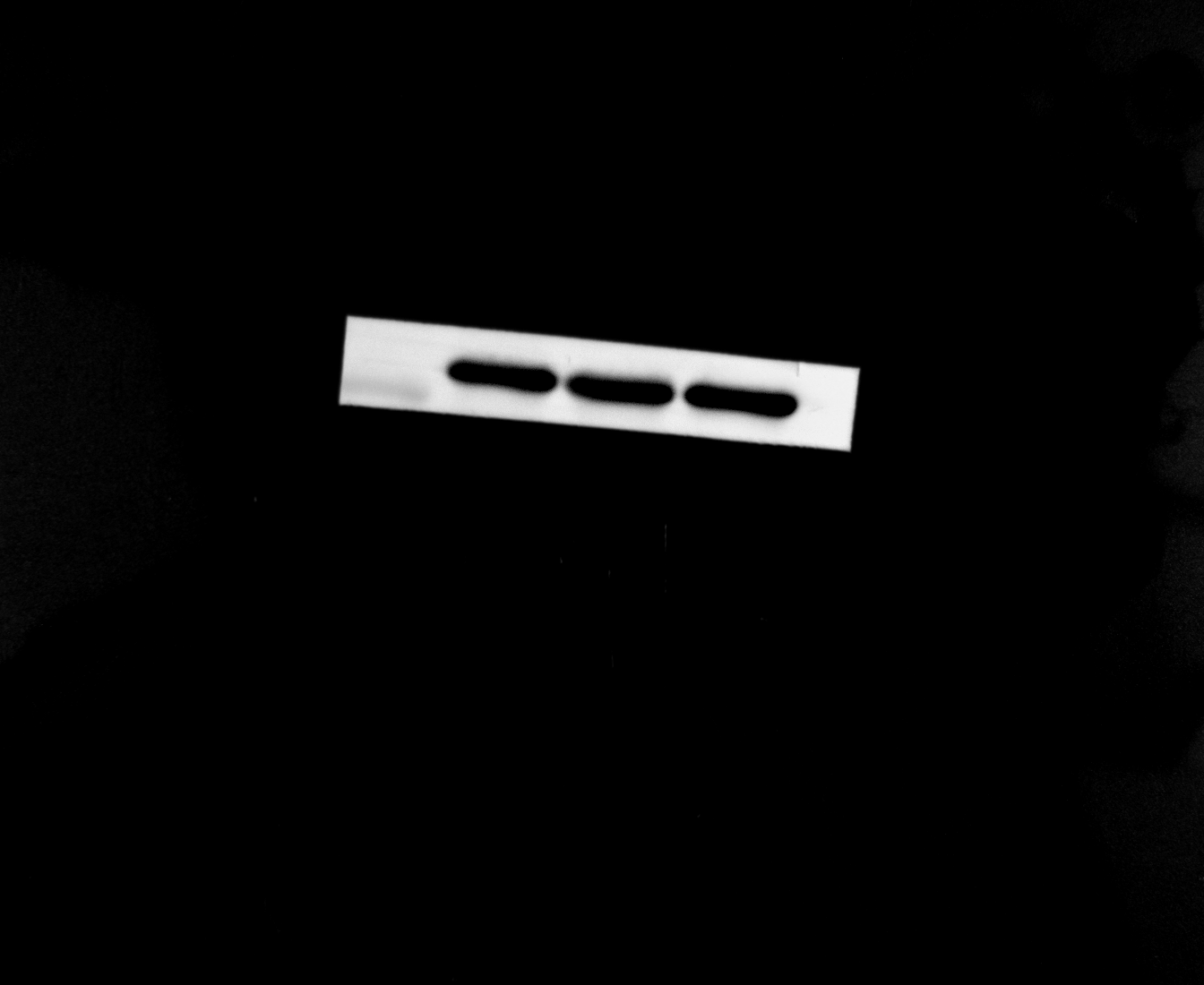

Supplement: Supplementary file 10 [file DataSheet7.ZIP › WB/2/gapdh.tif]

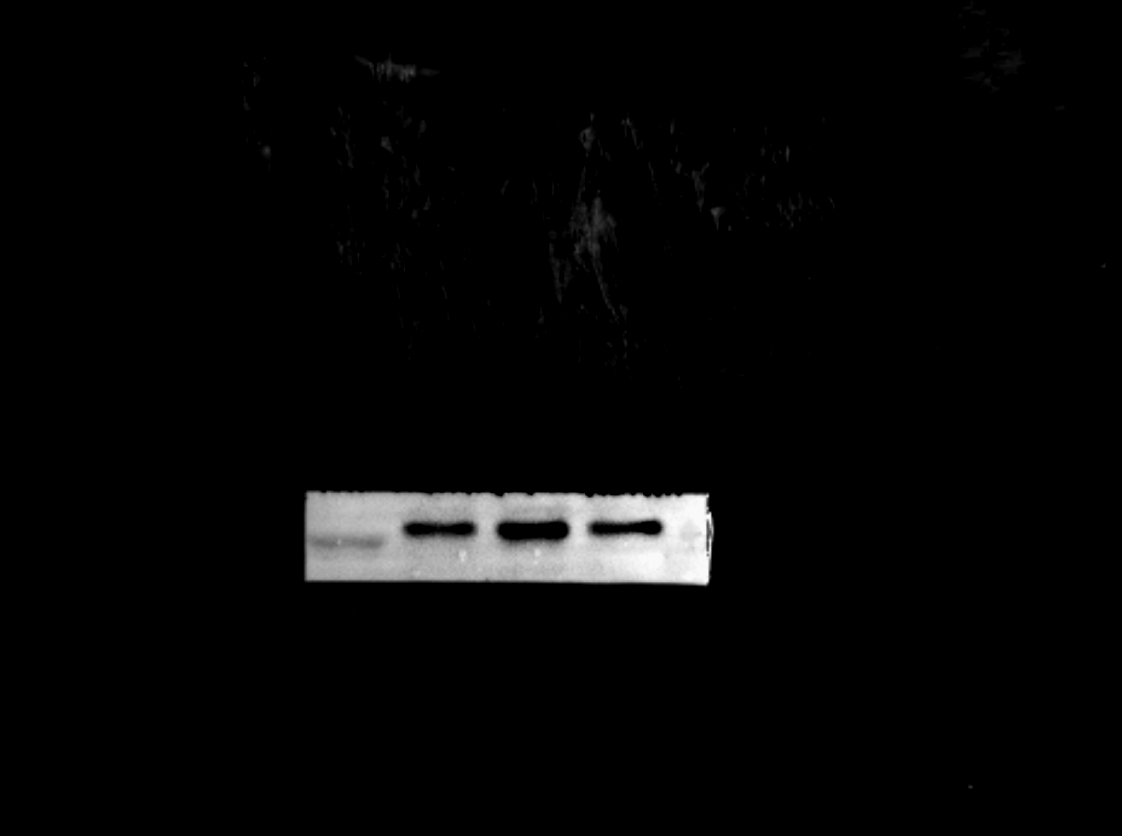

Supplement: Supplementary file 10 [file DataSheet7.ZIP › WB/2/HIF-1a┴.tif]

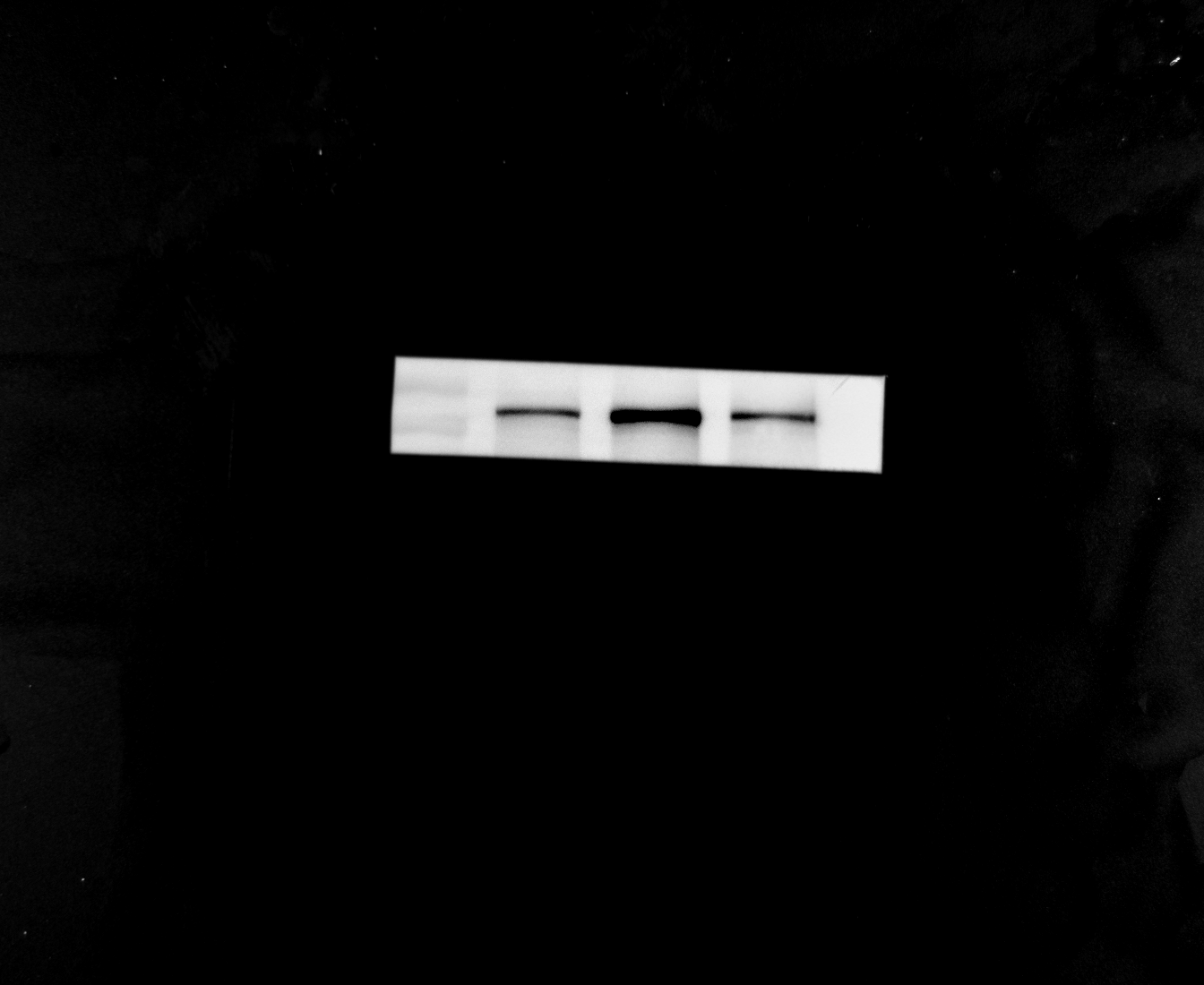

Supplement: Supplementary file 10 [file DataSheet7.ZIP › WB/2/HMOX1.tif]
