# Supplementary material for: Qingxuan Runmu Yin alleviates dry eye disease via inhibition of the HMOX1/HIF-1 pathway affecting ferroptosis
Source: Front Pharmacol. 2024 Sep 11;15:1391946. doi: 10.3389/fphar.2024.1391946 (PMC11425584; doi:10.3389/fphar.2024.1391946)
Supplement: Supplementary file 6 [file DataSheet1.PDF]

Supplementary Table 1. The intersection of disease targets with drug targets resulted

SOD1
MMP9
TNF
IL6
IFNG
PPARA
BCL2
PTGS2
CASP8
STAT3
ESR1
GPT
IL10
INS
ICAM1
IL13
NT5C1B
ADARB2
P2RY1
SARDH
ADRA1A
FCER2
ABAT
AMBP
PTGER3
ACTL6A
CHRM1
CCNB1
FASN
ACACA
P2RY11
MT3
IBSP
PSMD3
TRPA1
GRIA2
ADH1B
ADRA2A
ADRB3
SLC5A6
LCT
ADARB1
ACTL6B

MGAM
RIPK4
ACTG2
SLC2A4
PRKCB
PRSS1
HSD11B2
PKLR
CCNA2
PGR
SCNN1A
CYP1A2
CX3CL1
SI
SLC5A5
SLC6A2
OXA1L
MMP8
SOD3
ADRB1
POLD1
DPP4
AKR1B1
CYP2E1
TRPM4
CHI3L1
FGF7
CDK2
PTGS1
CASP9
ALOXE3
PRKCA
GHRL
ODC1
MAOA
ACTC1
CD86
SLC6A4
ADAR
ALOX5
MDM2
CYP19A1
HMGCR
RAC1

MAOB
BAX
NFKBIA
GCG
ATP2C1
TRPM1
PECAM1
KCNH2
JAK1
BCHE
CDKN1A
CASP1
HCRT
IL12A
DDC
NFKB1
SELE
JUN
ACHE
VCAM1
IL12B
ADA
CDK4
IRAK4
INSR
ADRB2
CASP3
CD40LG
CD79A
IGF2
KDR
PLAU
SLC6A3
EPO
FASLG
JAK2
CCND1
STAT1
EDN1
CTSD
NOS2
ADA2
MMP1
SREBF1

RB1
PON1
CYP1B1
IL2
HMOX1
AKT1
CDKN2A
SCN5A
MPO
CXCL8
NOS3
VEGFA
TP53
TYR
