# Supplementary material for: Qingxuan Runmu Yin alleviates dry eye disease via inhibition of the HMOX1/HIF-1 pathway affecting ferroptosis
Source: Front Pharmacol. 2024 Sep 11;15:1391946. doi: 10.3389/fphar.2024.1391946 (PMC11425584; doi:10.3389/fphar.2024.1391946)
Supplement: Supplementary file 9 [file DataSheet5.ZIP › apoptosis/apoptosis.pdf]

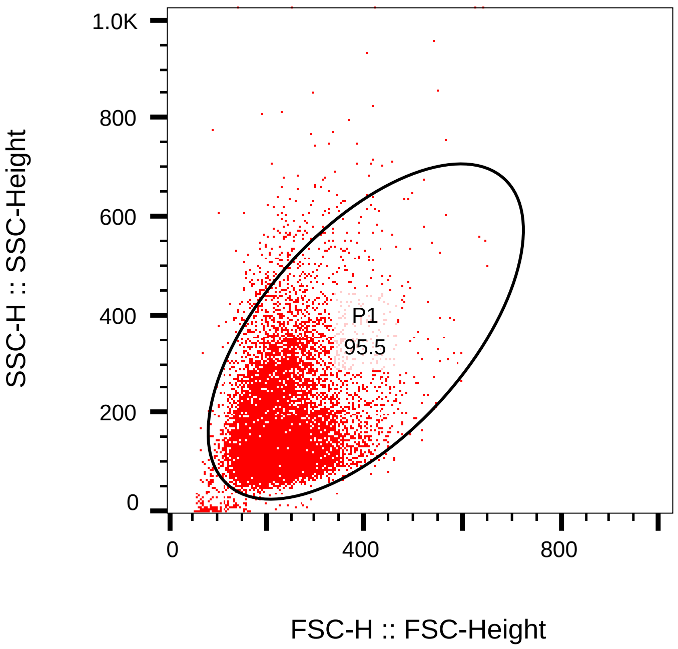

Data.325
Ungated
10000

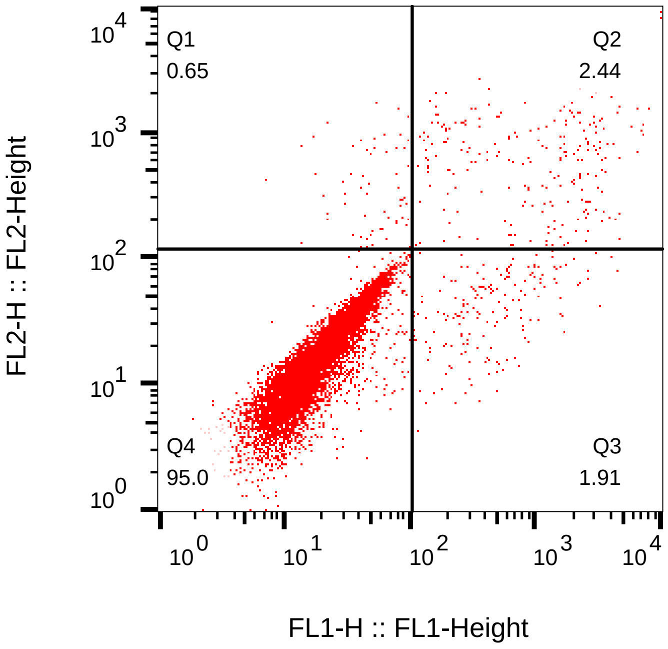

Data.325
P1
9548

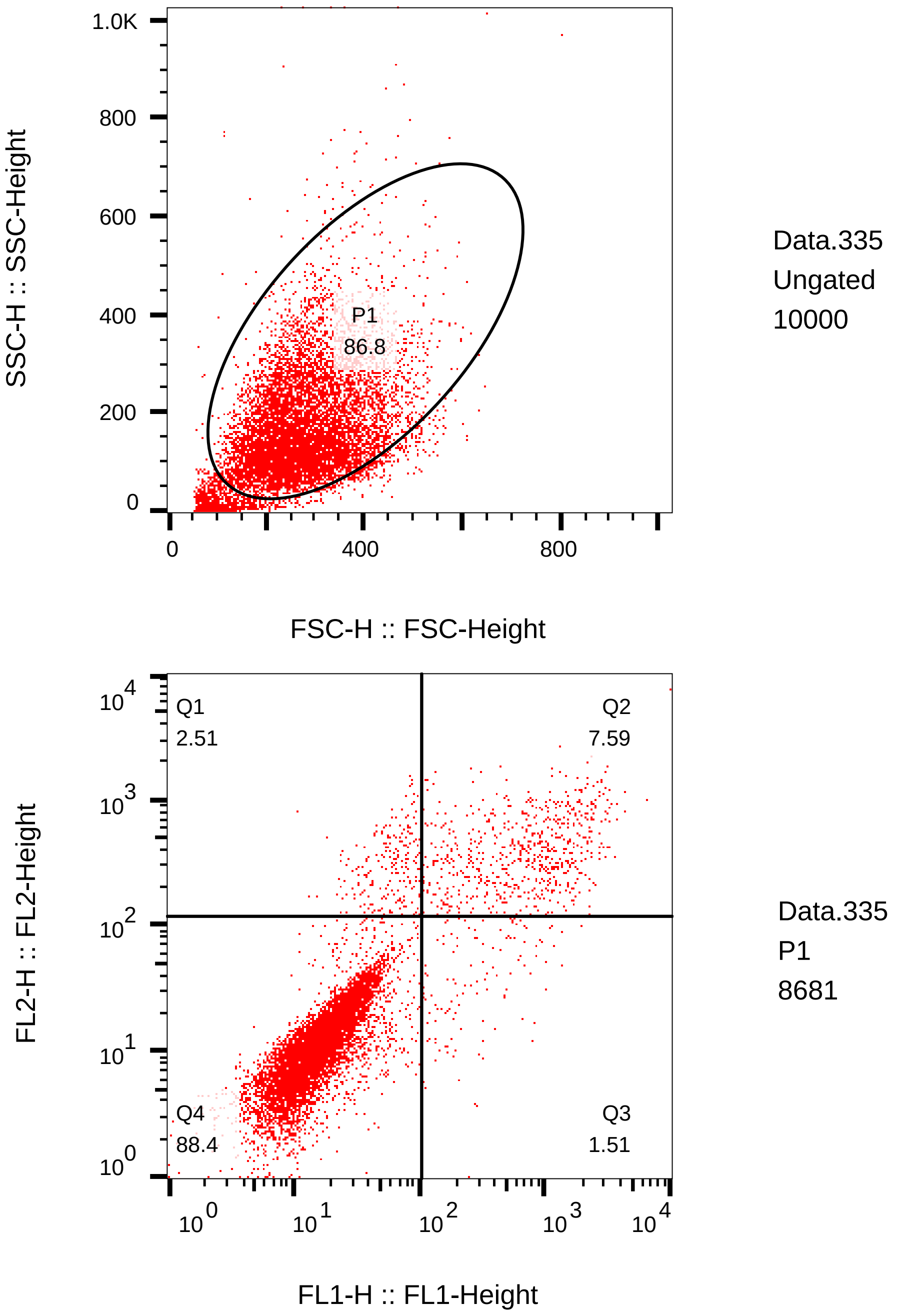
SSC-H :: SSC-Height
1.0K
800
600
400
200
0
P1
86.8
0
400
800
FSC-H :: FSC-Height
Data.335
Ungated
10000
FL2-H :: FL2-Height
Q1
2.51
Q2
7.59
Q4
88.4
Q3
1.51
10^0
10^1
10^2
10^3
10^4
FL1-H :: FL1-Height
Data.335
P1
8681

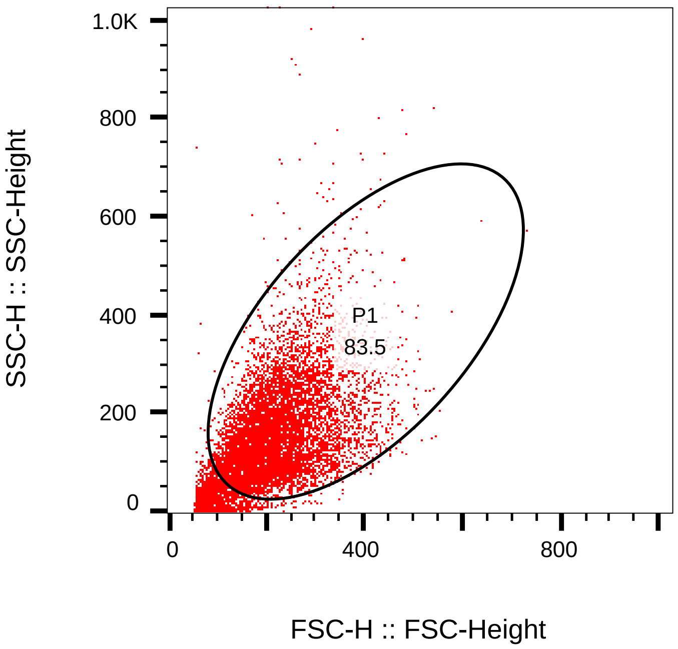

Data.376
Ungated
10000

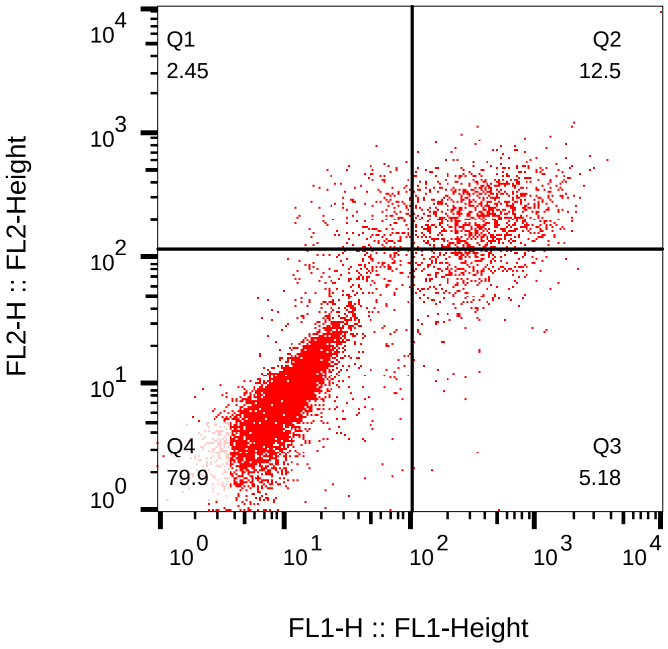

Data.376
P1
8353

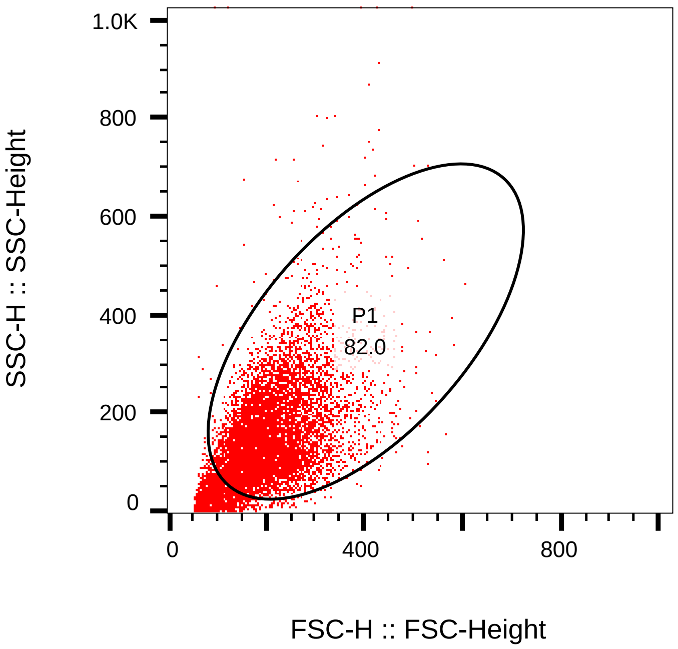

Data.385
Ungated
10000

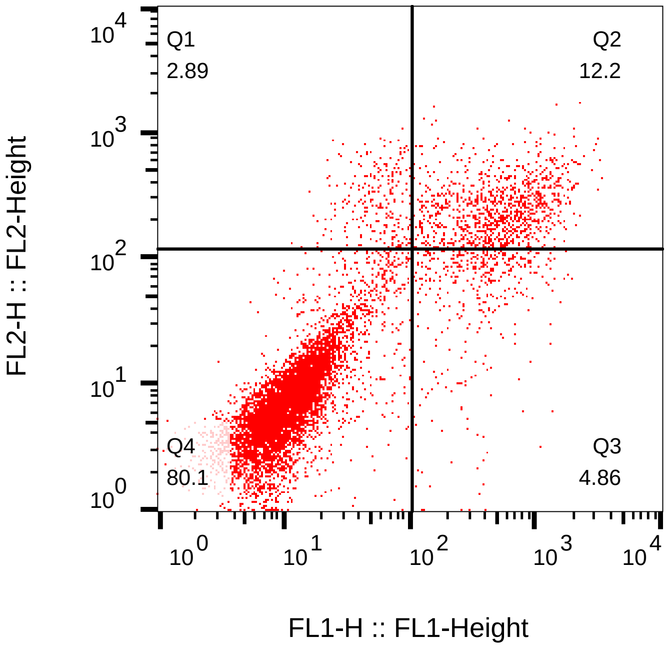

Data.385
P1
8195

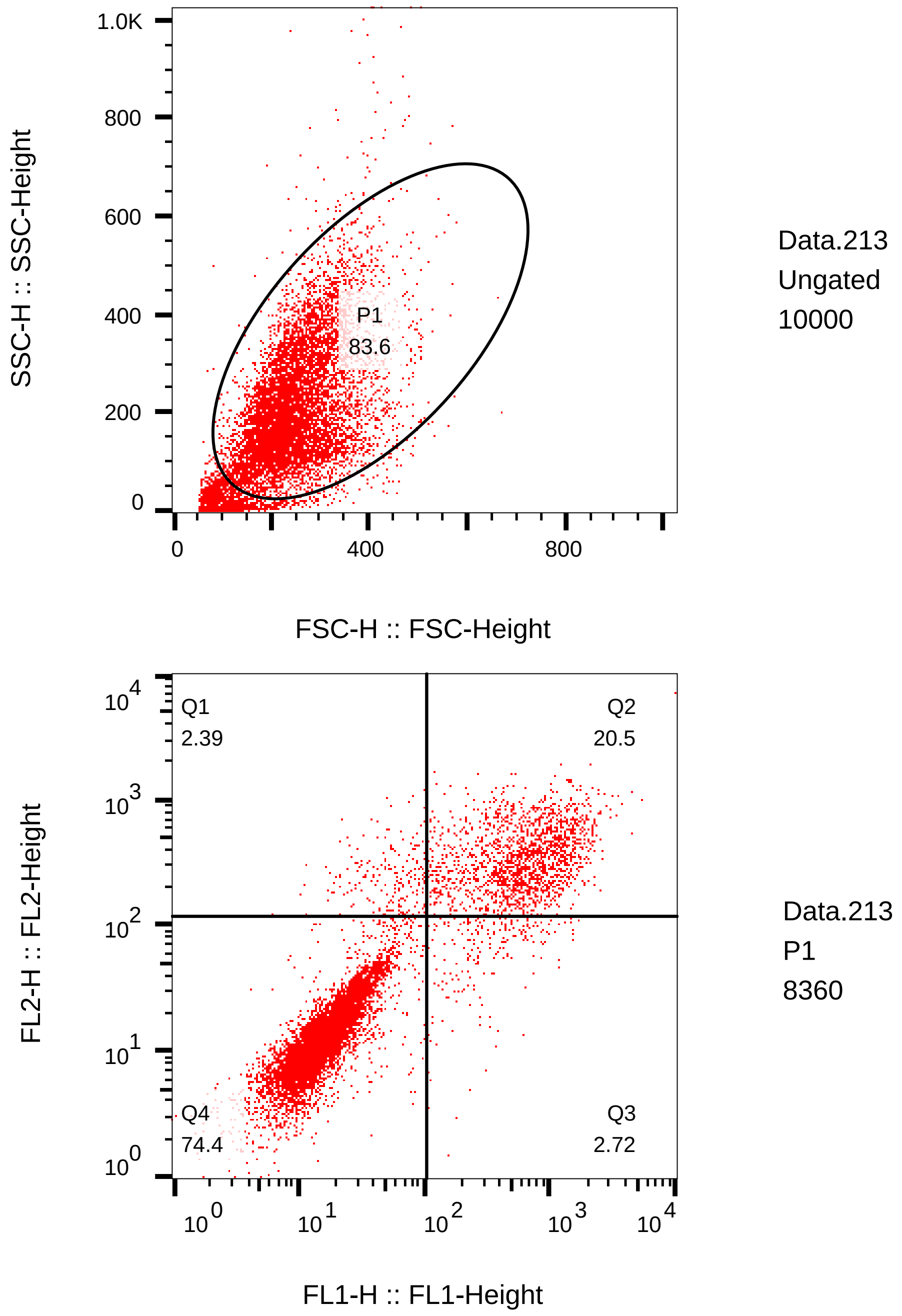
1.0K
800
600
400
200
0
SSC-H :: SSC-Height
P1
83.6
0
400
800
FSC-H :: FSC-Height
Data.213
Ungated
10000
Q1
2.39
Q2
20.5
Q4
74.4
Q3
2.72
FL2-H :: FL2-Height
FL1-H :: FL1-Height
Data.213
P1
8360

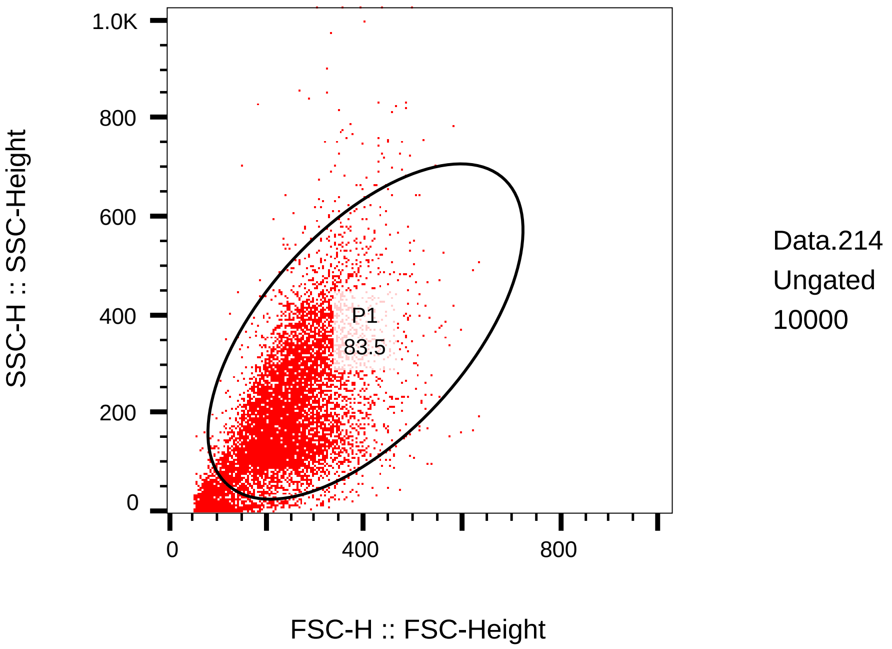

SSC-H :: SSC-Height
FSC-H :: FSC-Height
1.0K
800
600
400
200
0
0
400
800
P1
83.5
Data.214
Ungated
10000

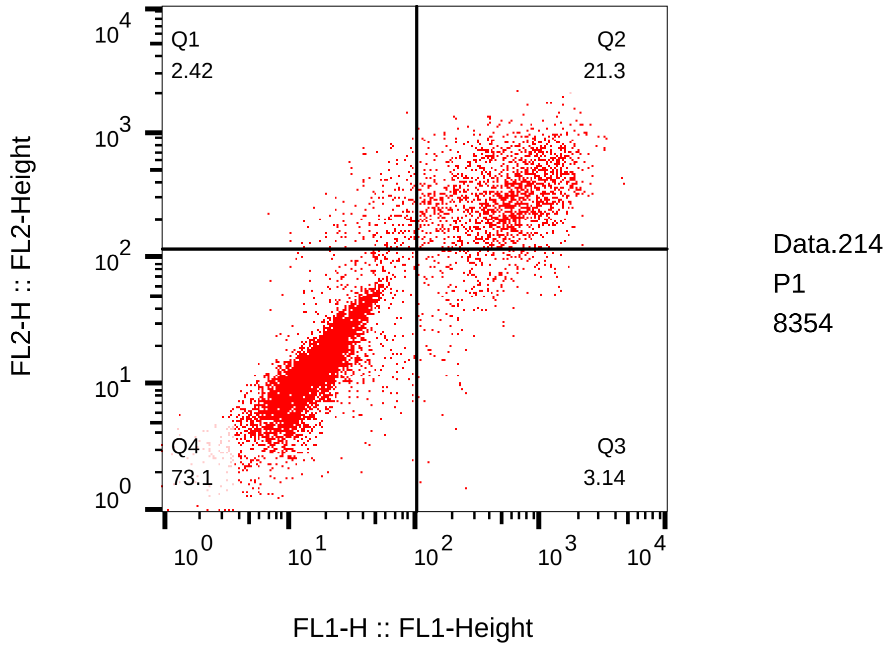

FL2-H :: FL2-Height
FL1-H :: FL1-Height
Q1
2.42
Q2
21.3
Q3
3.14
Q4
73.1
Data.214
P1
8354

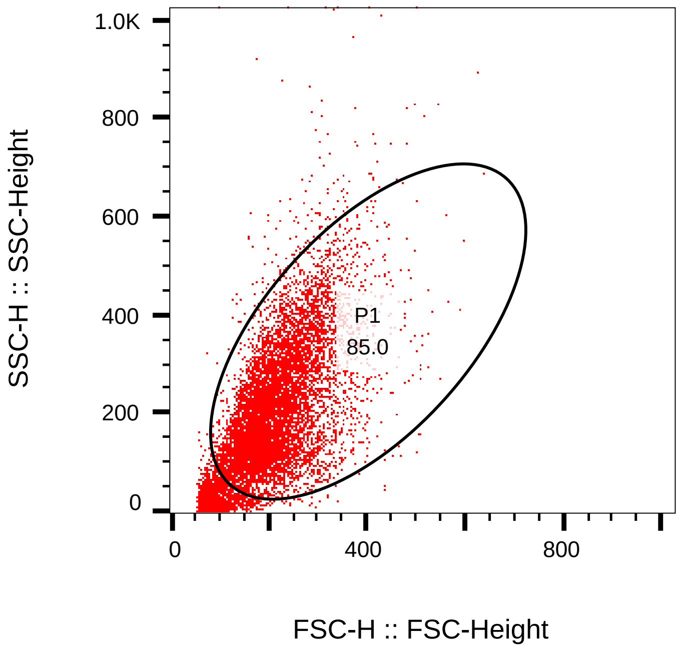

Data.243
Ungated
10000

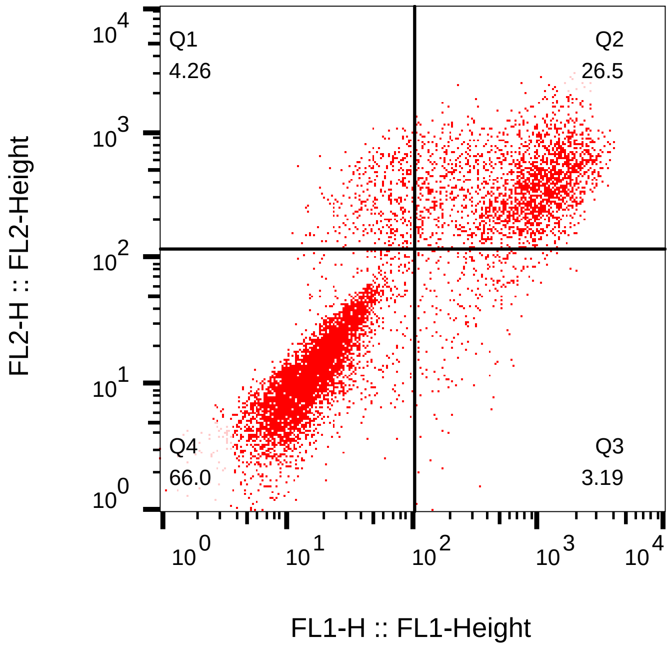

Data.243
P1
8500
